# Principal Component Analysis Based Feature Extraction Approach to Identify Circulating microRNA Biomarkers

**DOI:** 10.1371/journal.pone.0066714

**Published:** 2013-06-24

**Authors:** Y-h. Taguchi, Yoshiki Murakami

**Affiliations:** 1 Department of Physics, Chuo University, Tokyo, Japan; 2 Department of Hepatology, Graduate School of Medicine, Osaka City University, Osaka, Japan; Ospedale Pediatrico Bambino Gesù, Italy

## Abstract

The discovery and characterization of blood-based disease biomarkers are clinically important because blood collection is easy and involves relatively little stress for the patient. However, blood generally reflects not only targeted diseases, but also the whole body status of patients. Thus, the selection of biomarkers may be difficult. In this study, we considered miRNAs as biomarker candidates for several reasons. First, since miRNAs were discovered relatively recently, they have not yet been tested extensively. Second, since the number of miRNAs is relatively limited, selection is expected to be easy. Third, since they are known to play critical roles in a wide range of biological processes, their expression may be disease specific. We applied a newly proposed method to select combinations of miRNAs that discriminate between healthy controls and each of 14 diseases that include 5 cancers. A new feature selection method is based on principal component analysis. Namely this method does not require knowledge of whether each sample was derived from a disease patient or a healthy control. Using this method, we found that hsa-miR-425, hsa-miR-15b, hsa-miR-185, hsa-miR-92a, hsa-miR-140-3p, hsa-miR-320a, hsa-miR-486-5p, hsa-miR-16, hsa-miR-191, hsa-miR-106b, hsa-miR-19b, and hsa-miR-30d were potential biomarkers; combinations of 10 of these miRNAs allowed us to discriminate each disease included in this study from healthy controls. These 12 miRNAs are significantly up- or downregulated in most cancers and other diseases, albeit in a cancer- or disease-specific combinatory manner. Therefore, these 12 miRNAs were also previously reported to be cancer- and disease-related miRNAs. Many disease-specific KEGG pathways were also significantly enriched by target genes of up−/downregulated miRNAs within several combinations of 10 miRNAs among these 12 miRNAs. We also selected miRNAs that could discriminate one disease from another or from healthy controls. These miRNAs were found to be largely overlapped with miRNAs that discriminate each disease from healthy controls.

## Introduction

Specific and sensitive noninvasive biomarkers for the detection of human diseases, including malignancies, are urgently required to reduce worldwide morbidity and mortality caused by cancer [1]–[3]. Although some successful use of transcriptome components as biomarkers was reported [4], [5], circulating microRNAs have also recently been identified as new clinical biomarker candidates 6–12. MicroRNAs are post-transcriptional regulators that are involved in many physiological and pathophysiological conditions. A recent study by Keller *et al.* [13] compared the expression profiles of hundreds of blood-borne microRNAs across a variety of nonmalignant and malignant diseases to identify disease-specific expression patterns. The resulting microRNA expression data could be used to discriminate disease samples with a high level of accuracy, demonstrating the potential use of microRNA signatures for blood-based diagnosis of disease. Using extensive bioinformatics research, Keller *et al.* demonstrated that a wide range of cancers and other diseases could be discriminated from healthy controls by only miRNA expression. The data set Keller *et al.* used was the most extensive data set ever reported, i.e., it included various types of diseases (14 diseases plus normal controls) and a large number of patient (n = 384) and control (n = 70) data sets from a large number of blood-based miRNA biomarker studies.

In spite of this, selecting a biomarker based on feature-extraction techniques remains challenging. Although Keller *et al.* [13] successfully discriminated cancers and other diseases from healthy controls by using the expression of only 10 miRNAs (see Supplementary Table 6 on page 14 of their Supplementary Materials), they did not state which 10 miRNAs were selected because of the problem of stability, raised by Abeel *et al.* [14]. Stability is the measure of how stable feature selections are. For example, suppose we have 2 set of samples, each of which consists of 2 categories. When features are independently extracted so as to discriminate 2 categories for each sample, if the majority of selected features are common between the 2 samples, it can be considered a stable feature extraction. If not, it is unstable. That is, if selected features fluctuate depending on the sample, the stability of feature selection is poor. Conversely, if most features are selected independent of the sample, the stability of feature selection is high.

Although Keller *et al.* [13] employed 10-fold cross-validation, the selection of 10 miRNAs fluctuated between trials (see demonstration in the “Stability” subsection below). This prevented them from presenting 10 specific miRNAs as biomarkers to discriminate patients with cancers and other diseases from healthy controls. This is a significant disadvantage of their research if it is to be applied for clinical use, since it is impossible to decide in advance which miRNAs should be employed as biomarkers.

In order to overcome this problem, we propose a new feature selection technique to select miRNAs as biomarkers. This method is based on principal component analysis (PCA), more specifically, sparse PCA [15]–[19].

PCA [20] is a type of dimensional reduction or ordination analysis. Ordination analysis attempts to embed objects distributed in high dimensional space into lower dimensional space. In PCA, dimensional reduction is achieved by projection to lower dimensional space using linear transformation. Although PCA is a simple and classical method, it can often effectively reduce redundant information.

Sparse PCA is defined as follows. In contrast to ordinary PCA, which employs all features to express lower dimensional space, sparse PCA tries to express lower dimensional space by a smaller number of features, even if the accuracy decreases. That is, sparse PCA is a feature extraction method that eliminates unnecessary features through a method that is not uniquely defined, but varies depending on the implementation.

Some similar trials of this kind using clustering-based feature extraction have been reported [21], [22]. For example, Liu *et al*. [23], [24] proposed gene selection using spectral biclustering, Dy *et al* [25] used hierarchical clustering for feature selection of lung cancer image classification, and Modha *et al* [26] made use of k-means for feature extraction. However, these are feature selection methods that require prior knowledge of class partitions or labeling. At minimum, prior to feature selection, the previous methods require knowledge or inference of the number of clusters, which our current approach does not require. Moreover, there has been no discussion of the stability of feature selection when using these cluster-based feature selection criteria. Such stability problems have started to be discussed only very recently [14].

In contrast to both of these above-mentioned general feature extraction approaches and several previously proposed feature extraction methods especially designed for gene expression analysis, e.g., significance analysis of microarrays (SAM) [27], gene selection based on a mixture of marginal distributions (gsMMD) [28], and ensemble recursive feature elimination (RFE) [14], our approach is free from stability problems. These types of classification-independent and stability-problem-free approaches were invented only very recently (e.g., unsupervised feature filtering (UFF) [29]) and are still very rare.

For each pair of diseases and normal controls, our approach enabled the selection of a set of 10 strict (confident) miRNA biomarker candidates. Each set of miRNAs could not only accurately discriminate patients with each disease from normal controls, but could also accurately discriminate one disease from another. Moreover, most of the sets shared the majority of miRNAs, which would allow for simplification of the measurement of a biomarker since a limited number of sets of miRNA measurements would permit the discrimination of several diseases.

The reason why we tried to employ blood-based biomarkers in spite of the drawbacks that miRNAs in blood inevitably reflect the whole body status and thus have less of a relationship with targeted diseases is because previous studies have demonstrated that the use of several circulating miRNAs can work well in a practical sense. Thus unification and consideration of our analysis results will improve the ability of miRNAs as biomarkers.

## Materials and Methods

### Feature Extraction Methods

#### PCA-based feature extraction

Suppose we have the miRNA profiles 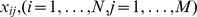, each corresponding to the 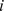
th miRNA in the 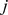
th sample. 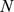 and 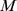 are the total number of miRNAs and samples, respectively. Samples were classified into 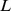 clinical sets, 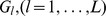. We applied PCA to the 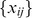 set in 2 ways:

1. Method 1 (miRNA-based): Substitute 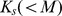 principal component (PC) score 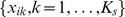 to 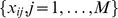. In this case, PCA was applied to a matrix 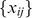.
2. Method 2 (sample-based): Substitute 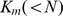 PC score 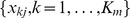 to 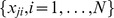. In this case, PCA was applied to a transverse matrix 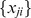.

PCA-based feature extraction was performed as follows.

1. Choose a pair of clinical sets, 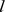 and 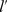.
2. Compute 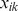 with Method 1 PCA from 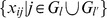.
3. Compute distance 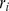,

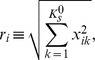



where 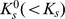 is the number of components to be used for feature selection.

1. Select miRNAs 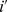 with top 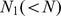 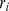
s.

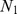
 miRNAs are a set of selected features to distinguish clinical sets 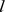 and 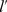. Throughout this paper, 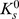 was assumed to be 2 unless stated otherwise. PCA was computed by the prcomp function in the R base package [30].

It should be noted that our method did not use any classification information. This enabled us to obtain stable feature extractions.

#### t-test based feature extraction

The 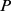-value for the 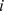
th miRNA between 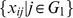 and 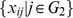 was computed using a 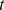 test. The top 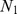 miRNAs with smaller 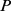-values were selected.

#### SAM-based feature extraction

The 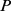-value for the 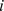
th miRNA between 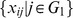 and 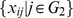 was computed using SAM. The top 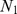 miRNAs with smaller 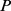-values were selected.

#### gsMMD-based feature extraction

Significantly up- or downregulated miRNAs were selected by gsMMD, which was implemented in Bioconductor software. The 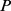-values for up- or downregulation were considered separately, and the top 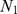 miRNAs were selected for both up- and downregulated miRNAs.

#### RFE- and ensemble RFE-based feature extraction

As described by Abeel *et al.* [14], the support vector machine with a linear kernel was applied to 40 independent resampled sets (with replacements) for ensemble RFE. After 100 independent cross-validations with 10% test samples and 90% training samples, the top 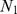 miRNAs with better accuracy were extracted. For simplicity, we employed only complete linear aggregation for weighting among each resampled set. No resamplings were conducted for simple RFE, and only cross-validations were performed. The top 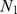 miRNAs with better accuracy were selected.

#### UFF

As described by Varshavsky *et al.* [29], differential singular value decomposition (SVD)-entropy 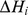,
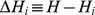


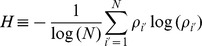


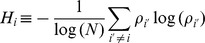


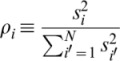
where 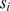 is a singular value and 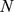 is the total number of miRNAs, was attributed to each miRNA. After 100 independent cross-validations with 10% test samples and 90% training samples, the top 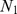 miRNAs with larger 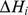 were selected.

### PCA-based Linear Discriminant Analysis (LDA)

PCA-based LDA was conducted as follows:

1. Choose a pair of clinical sets, 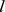 and 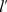.
2. If necessary, apply feature extraction and reduce the number of miRNAs used for LDA.
3. Compute 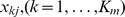 using Method 2 PCA.
4. Divide samples into training and test sets.
5. Apply LDA to training set.
6. Validate performance of LDA using test set.
7. Repeat steps 4–6 the specified number of times depending on the employed cross-validation method.
8. Compute performance with averaged values.
9. Estimate the optimal value of 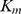 by repeating steps 3–8 as 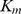 changes.

It should be noted that the division between training and test sets was carried out **AFTER** the computation of PCA (and feature extraction if necessary). Thus, 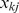 includes the test set information as well. Feature extraction, if applied, was also conducted before division was performed; thus, it was sampling free. One may suggest that this was erroneous since we do not know the classification of the test set. However, we can compute the PCA even if we do not have prior knowledge of the classification because we do not need classification information to compute 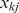. This is explained in the “Why did PCA-based feature selection work so well?” subsection in Results and Discussion section as well. The LDA was computed by the lda function in the R base package [30].

### miRNA Expression and Normalization

The miRNA expression used in this study was obtained from the Gene Expression Omnibus (GEO) accession number GSE31568, which was used by Keller *et al.* [13]. We downloaded GSE31568_raw and normalized miRNA expression within each sample to obtain the mean and standard deviation (SD).

### Stability Test

As in Abeel *et al.* [14] and Varshavsky *et al* [29], we evaluated whether the selection of miRNAs for the discrimination between patients with diseases and healthy controls was stable [13]. The procedure was as follows:

1. Choose a pair of clinical sets, each including one cancer or other disease sample and one healthy control sample.
2. Pick 90% samples randomly, independent of classification.
3. Apply feature selection to select 10 miRNAs as biomarkers.
4. Repeat steps 2 and 3 a total of 100 times and count the frequency of each miRNA selection.
5. Repeat steps 2–4 for all pairs of diseases and healthy controls.

The above procedures were applied to all feature extraction methods, i.e., those based on 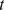-tests, PCA, SAM, gsMMD, RFE, RFE ensemble, and UFF.

### Amount of Contribution from each miRNA to Discrimination

Suppose we obtained 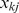 by PCA analysis after PCA-based feature extraction was applied. Then
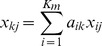



If we applied LDA to discriminate one cancer or disease from one healthy control using 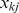, we obtained the discriminant function 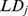 as
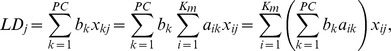
for the 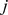
th sample, where 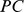 is the number of 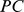
s used for discrimination. Typically, a positive (negative) 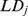 indicates that the sample 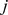 represents a patient with cancer or another disease (healthy control) sample. Then, the amount of contribution, 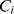, of miRNA 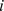 to the discriminant function is



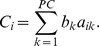



### KEGG Pathway Analysis of miRNA Target Genes

#### DIANA-mirPath

DIANA-mirPath [31] is a web tool. We used this software version for the implementation of multiple miRNAs (http://diana.cslab.ece.ntua.gr/pathways/index_multiple.php). DIANA-mirPath accepts a set of miRNAs, estimates union of miRNA target genes, and finally computes 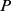-values that describe KEGG pathway enrichment of the target genes. For the data set of our selection, we extracted up- or downregulated sets of miRNAs and uploaded them onto DIANA-mirPath. Using default settings, DIANA-mirPath employed a list of target genes estimated by DIANA-microT v4.0. When we needed to infer 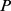-values attributed to KEGG pathways for other studies, we uploaded a set of miRNAs identified as biomarkers in the relevant research.

#### Starbase

Starbase [32] is another web tool. Among several tools provided by Starbase, we used miRPathway(http://starbase.sysu.edu.cn/miRPathway.php) to infer 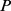-values attributed to KEGG pathways. Instead of target gene tables inferred computationally, Starbase employed cross-linking immunoprecipitation (CLIP)-Seq data. This has both advantages and disadvantages. One advantage was the certainty of the miRNA target, while one disadvantage was the range of targets. If some genes are listed as targets of some miRNAs, it is very likely true. On the other hand, if no CLIP-Seq data exists for targeted diseases, there are likely no disease-specific miRNA target genes listed. Thus, we employed Starbase to support mirPath software. The lack of detection of KEGG pathways listed by mirPath is not discussed here. All parameters were kept as default values.

## Results and Discussion

### Simulation

Before performing biomarker selection of miRNAs for real data sets, we performed numerical simulations that compared our proposed method with 2 other methods, i.e., SAM-based and 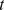-test based feature extraction (for details, see 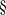
1 Simulation in Text S2). In this simulation, we prepared 100 miRNAs with 200 samples. The first 100 samples belonged to category 1, while the second 100 samples belonged to category 2. Among 100 miRNAs, only the first 10 miRNAs exhibited distinct expression between the 2 categories. The task is to select 10 correct miRNAs among the 100 miRNAs and achieve better performance for the discrimination between the 2 categories. We tested 3 scenarios. In the scenario I, expression differences of the 10 miRNAs were kept constant, while noise added to these 10 miRNAs was varied. In scenario II, the expression differences of the 10 miRNAs varied, while the noise added to these 10 miRNAs was kept constant. In scenario III, both expression differences of the 10 miRNAs and noise added to the 10 miRNAs were varied simultaneously (Table 1, for more detailed discussion, see Text S2). Then, we found that our method outperformed the other 2 methods over a wide range of parameters. Thus, we concluded that our method can achieve both better performance of discrimination and more ability to select features that differ between the 2 categories. R core that generates simulation data set used in this study can be found in Text S3.

**Table 1:** Performance of several feature extraction methods for scenario III.

		Accuracy			# of miRNAs		
*D_μ_*	*D_σ_*	*t* test	PCA	SAM	*t* test	PCA	SAM
2.0	0.5	0.99	0.99	0.99	9.0	8.7	8.0
2.0	1.0	0.95	0.95	0.95	8.1	7.9	7.5
2.0	1.5	0.88	0.88	0.88	7.7	9.2	7.5
2.0	2.0	0.83	0.82	0.82	6.8	9.3	6.9
1.5	0.5	0.98	0.98	0.98	8.7	8.0	7.2
1.5	1.0	0.90	0.88	0.90	7.9	8.0	7.1
1.5	1.5	0.82	0.81	0.82	7.1	8.5	6.7
1.5	2.0	0.77	0.76	0.77	6.4	8.9	6.5
1.0	0.5	0.95	0.89	0.94	8.2	6.2	6.5
1.0	1.0	0.82	0.75	0.81	6.9	6.4	6.0
1.0	1.5	0.71	0.66	0.71	6.2	7.1	5.9
1.0	2.0	0.66	0.63	0.66	5.4	8.1	5.4
0.5	0.5	0.82	0.50	0.80	6.9	0.0	4.5
0.5	1.0	0.66	0.50	0.65	5.3	0.1	3.9
0.5	1.5	0.59	0.50	0.57	3.9	2.1	3.5
0.5	2.0	0.55	0.51	0.55	3.5	4.4	3.4

Accuracy and the number of correctly selected miRNAs among 10 miRNAs with distinct expression between the 2 classes (averaged over 100 trials) for *t*-test-, PCA-, and SAM-based feature extractions. Scenario III was employed. Upper rows indicate easier classification problems. 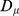 and 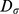 represent the amplitudes of mean and standard deviation of the first 10 miRNAs that exhibit distinct expression between the 2 categories.

### Biomarker Identification for the Discrimination of Patients with Cancers and other Diseases from Healthy Controls

Based on the findings in the previous section, we employed and applied PCA-based feature extraction to biomarker identification for cancers and other diseases [13]. As we will explain in the “Why did PCA-based feature selection work so well?” subsection in this section, since our PCA-based feature extraction was free from sampling, we could strictly define the top 10 miRNAs that were distinct between pairs of clinical samples and healthy control samples (Table 2). The reasons we employed 10 miRNAs as biomarkers were as follows. First, a previous study [13] extensively studied a situation in which 10 miRNAs were employed as biomarkers, making it easy for us to compare our findings with their discrimination performances, e.g., accuracy, sensitivity, and specificity. Second, as can be seen below, using 10 miRNAs as biomarkers allowed us to achieve sufficiently good performance. Third, measurement of 10 miRNAs is practical for clinical use. Finally, as stated in the previous section, 10 miRNAs were sufficient to achieve a performance comparable to that of all (100) miRNAs.

**Table 2:** miRNAs selected to distinguish patients with cancers or other diseases from healthy controls by PCA-based feature extraction.

		lung cancer		other pancreatic tumors and diseases	pancreatitis	ovarian cancer	COPD (chronic obstructive pulmonary disease)	ductal pancreatic cancer	gastric cancer	sarcoidosis	prostate cancer	acute myocardial infarction	periodontitis	multiple sclerosis	melanoma	Wilm’s tumor
A	hsa-miR-425	+	+		+	−	+	+	−	+	+	−	−	−	−	−
A	hsa-miR-191	+	+		+	−	+	+	+	-	+	−	−	*	+	−
	hsa-miR-185	−	−		−	−	−	−	−	+	−	−	−	−	+	−
	hsa-miR-140-3p	+	+		+	+	+	−	+	+	+	−	+	+	+	+
B	hsa-miR-15b	−	−		−	+	+	+	−	−	−	+	−	−	−	+
B	hsa-miR-16	−	+		+	+	+	+	+	−	+	−	+	+	−	−
	hsa-miR-320a	+	−		−	−	+	−	−	+	+	+	+	+	+	+
	hsa-miR-486-5p	−	+		+	−	−	+	−	+	−	+	−	−	−	−
C	hsa-miR-92a	±	+		−	+	−	±	+	+	−	+	−	+	±	+
C	hsa-miR-19b	±	*		*	*	*	±	*	*	*	*	*	-	±	*
	hsa-miR-106b	*	+		+	−	+	*	−	*	−	−	−	−	*	−
	hsa-miR-30d	*	*		*	*	*	*	*	+	*	*	*	*	*	*

+ (−) indicates that the miRNA was expressed in patients with cancers or other diseases (healthy controls).

* indicates that the miRNA was not selected within the top 10 most significant miRNAs contributing to discrimination. A–C: miRNAs belonging to common clusters, which were defined by an inter-miRNA distance of 1 kbp. Coincidence within clusters A and C are underlined. See Fig. 5 for actual amount of expression/suppression.

We can also make use of these 10 selected miRNAs for discrimination between patients with diseases and healthy controls. The performance of PCA-based LDA between patients with diseases and healthy controls using only these 10 miRNAs is summarized in Table 3. In contrast to Keller *et al.* [13], we successfully identified 10 miRNAs as biomarkers. Keller *et al* could not do this because 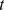-test-based feature extraction is highly dependent on divisions between training and test sets. Since they carried out 100 division trials, it would have been impossible for them to create a definite set of 10 miRNAs (see the “Stability” subsection in this section).

**Table 3:** Performance of PCA-based LDA for discrimination between patients with cancers or other diseases and healthy controls.

cancer orother disease	PC	Accuracy	Specificity	Sensitivity	Precision
Lung cancer	5	0.784 (+)	0.800 (+)	0.750 (+)	0.632
Other pancreatic tumors and diseases	7	0.814 (+)	0.771	0.875 (+)	0.724
Pancreatitis	8	0.833 (+)	0.786 (−)	0.921 (+)	0.700
Ovarian cancer	6	0.800	0.786 (−)	0.867 (+)	0.464
COPD	2	0.713 (−)	0.671 (−)	0.833 (+)	0.465
Ductal pancreatic cancer	2	0.765 (−)	0.743 (−)	0.800 (+)	0.667
Gastric cancer	9	0.855 (+)	0.857 (+)	0.846	0.524
Sarcoidosis	10	0.835 (−)	0.800 (+)	0.889 (−)	0.741
Prostate cancer	5	0.806 (+)	0.800 (+)	0.826 (+)	0.576
Acute myocardial infarction	7	0.789 (−)	0.900	0.757 (−)	0.964
Periodontitis	10	0.807 (+)	0.814 (+)	0.778 (−)	0.519
Multiple sclerosis	10	0.892 (+)	0.871 (+)	0.957 (+)	0.710
Melanoma	10	0.867 (−)	0.857 (+)	0.886 (−)	0.756
Wilm’s tumor	7	0.867	0.886	0.600	0.273

+ (−) indicates that the performance was better (worse) than that of Keller *et al* [13]. PC is the number of PCs used for PCA-based LDA. LOOCV was applied. See the Table in the study by Keller *et al* [13] on page 14 of the Supplementary Materials.

**Figure 1:**
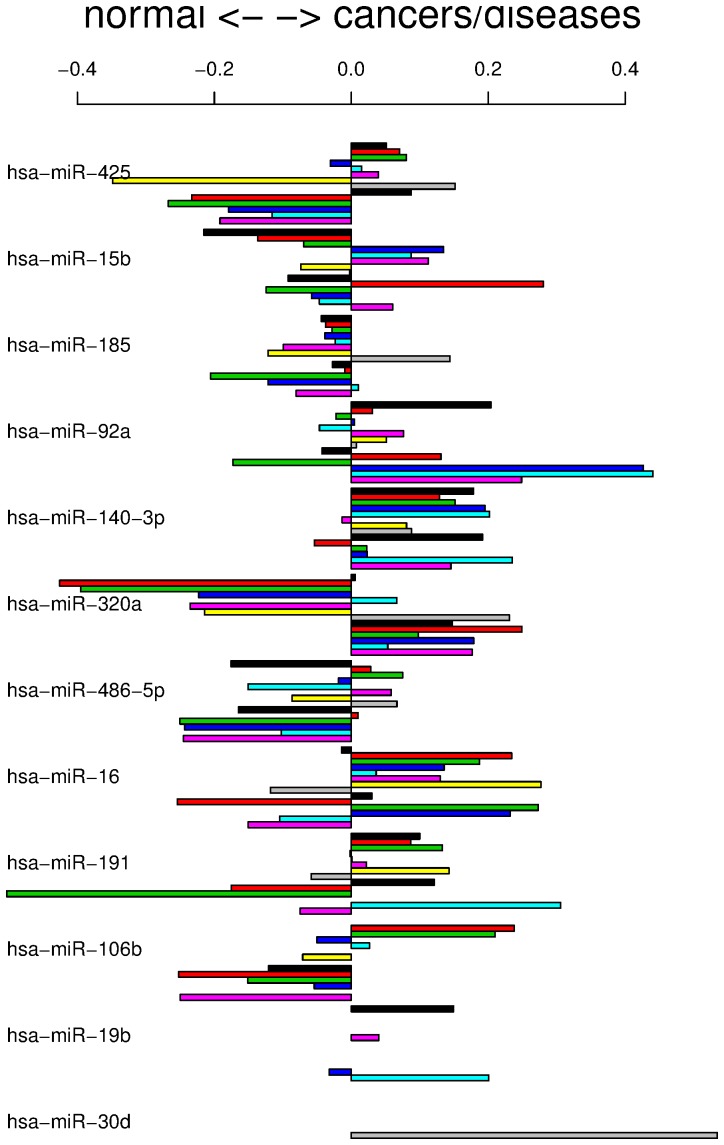
Individual contributions of miRNAs to discrimination between patients with cancers or other diseases and normal controls. The height of the bars indicates the amount of contribution from each miRNA in discriminating patients with cancers or other diseases from healthy controls. A positive (negative) value indicates that the miRNA was expressed in patients with cancer and other diseases (healthy controls). The order of cancers or other diseases is the same as that in Table 2 (top to bottom): lung cancer (black), other pancreatic tumors and diseases (red), pancreatitis (green), ovarian cancer (blue), COPD (cyan), ductal pancreatic cancer (pink), gastric cancer (yellow), sarcoidosis (grey), prostate cancer (black), acute myocardial infarction (red), periodontitis (green), multiple sclerosis (blue), melanoma (cyan), and Wilm’s tumor (pink).

Instead of a list of 10 miRNAs used for discrimination, they listed miRNAs that were deregulated in at least 6 diseases (Keller *et al.* [13] Supplementary Table 2). Surprisingly, there was very little overlap between the miRNAs reported by Keller *et al* and the miRNAs reported in our Table 2 in the present study. In fact, the only overlapping miRNA was hsa-miR-16. Even if we took Figure 1 in the study by Keller *et al*. [13] into account, where upregulated miRNAs were considered together, no other miRNAs were selected both in their paper and in the present study.

Recently, Keller *et al.* [33] attempted similar research with next-generation sequencing. They renewed a list of significant miRNAs in the supplementary information of their original study, but again, there were only 2 overlaps with the current study, i.e., miR-425 (for gastric cancer and Wilm’s tumor) and miR-140-3p (for melanoma, ovarian cancer, and periodontitis).

#### Comparison with previous studies

In order to validate our selections independent of the research by Keller *et al*, we have reviewed the literature for previous reports to support our findings that these miRNAs are closely related to cancers and other diseases. We discovered a large number of previously published reports supporting the relationship between diseases and the miRNAs observed in this study (Table 2 and Text S1). Although the reports were not always consistent, miR-15b, miR-185, miR-140-3p, miR-320a, miR-486-5p, miR-16, and miR-30d were found to function generally as tumor suppressors, and miR-425, miR-92a, miR-191, miR-106b, and miR-19b were primarily oncogenic. In order to confirm the validity of our evaluation, we listed the reported up- and downregulated miRNAs in several cancers in Table 4. However, since not all miRNAs have been reported to be up- or downregulated, the fact that most of the miRNAs in Table 2 were also included in Table 4 (with the exception of miR-320, miR-486, and miR-191) supports the notion that our findings agree with those of previous studies. Their up- and downregulation patterns are essentially consistent with what we have described above, since a tumor suppressor (oncogene) should be suppressed (expressed) in cancers. Among these, some miRNAs exhibited slightly more complicated functionalities. For example, miR-185 was frequently upregulated in cancers (see Table 4) while its expression sometimes suppressed cell proliferation (see Text S1). Another example of an miRNA with complicated features is miR-15b, which was not always suppressed in tumors. As shown in Table 4 this miRNA was upregulated in colon cancer, but sometimes inhibited tumor function (see Text S1). This somewhat difficult-to-understand situation can be observed in expression profiles as well. Even when reviewing a heat map (Fig. S1), one can discern that no specific expression of miRNAs was associated with cancers and other diseases. Thus, we need to develop approaches that are more sophisticated than observing individual miRNA expression one at a time.

**Table 4:** miRNAs in Table 2 whose up- and/or downregulation in any cancer was reported in the study by Bandyopadhyay *et al.* [41].

miRNA	Cancer type	Expression	Mean fold change
hsa-miR-425	Central nervous system	Downregulated	13.6-fold reduction
hsa-miR-15b	Colon	Upregulated	1.5-fold increase
hsa-miR-185	Bladder (urothelial)	Upregulated	1.30-fold increase
hsa-miR-185	Kidney	Upregulated	1.42-fold increase
hsa-miR-92-2	Pancreas	Upregulated	
hsa-miR-92-2	Prostate	Upregulated	
hsa-miR-140	Central nervous system	Downregulated	2.7-fold reduction
hsa-miR-140	Colon	Downregulated	11.4-fold reduction
hsa-miR-140	Hematologic	Downregulated	3.5-fold reduction
hsa-miR-140	Lung	Downregulated	
hsa-miR-140	Ovary	Downregulated	3.51-fold reduction
hsa-miR-16-1	Uterus/endometrial _cancer	Upregulated	At least 2-fold increase
hsa-miR-16-2	B cell CLL	Downregulated/Deleted	
hsa-miR-16a	B cell CLL	Downregulated	
hsa-miR-191	Breast	Upregulated	
hsa-miR-191	Central nervous system	Downregulated	4.4-fold reduction
hsa-miR-191	Colon	Upregulated	1.4-fold increase
hsa-miR-191	Lung	Upregulated	
hsa-miR-106b	Lung	Upregulated	12-fold increase in small lung cancer cell line SKLC-2.
hsa-miR-30d	Central nervous system	Downregulated	3.2-fold reduction

Any miRNAs listed in the Additional File in the study by Bandyopadhyay *et al.* [41].

#### Probability of different disease sharing same miRNA subsets

If we also consider the fact that our list was common for most of the comparisons between healthy control samples and disease samples, we believe that our list of miRNAs as biomarkers to distinguish between patients with cancers or other diseases and healthy controls was accurate. Such a trend would rarely occur only because of simple accidental/coincidental agreement; there are too many miRNAs for this to occur by chance. Suppose that there are 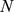 miRNAs and we select 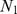 among them. Assuming the selection of 10 miRNAs as biomarkers from a total 862 miRNAs are independent of each other, the expected number of miRNAs being always selected for 14 selections is 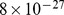, when 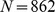 and 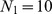. This is much less than the number of common miRNAs in Table 2, i.e., 8 (miR-425, miR-15b, miR-185, miR-92a, miR-140-3p, miR-320a, miR-486-5p, and miR-16). Thus, our list is plausible even if it does differ dramatically from Supplementary Table 2 reported by Keller *et al.* [13]. Nevertheless, there are no theoretical/biological reasons that a set of 10 representative miRNAs used to discriminate between patients with cancers or other diseases and healthy controls must be unique.

#### Disease-specific co-expression of miRNAs

In order to understand more deeply how each miRNA cooperatively discriminates between cancers or other diseases and healthy controls, we visualized the contribution of each miRNA to discrimination (Fig. 1 and Table 2). Since LDA is a linear method, it allowed us to do this easily (see Materials and Methods).

Interestingly, miRNAs that belong to the same cluster, defined by a inter-miRNA distance of 1 kbp, often share combinations of positive/negative contributions. For example, in Table 2, there are remarkable coincidences between miR-92a and miR-19b in the rows labeled “C” in the left column and those in the same row that are underlined. Three (lung cancer, ductal pancreatic cancer, and melanoma) out of 4 cancers or other diseases for which contributions of miR-19b are listed shared the same outcomes, although they were not significant (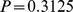). Similarly, miR-425 and miR-191 (rows “A” and underlined in the same row in Table 2) had the same positive/negative contributions for 10 (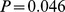) out of 13 cancers or other diseases, whereas miR-191 made non-zero contributions (3 exceptions: gastric cancer, sarcoidosis, and melanoma). However, since this does not hold true for miR-15b and miR-16 (rows “B” but not underlined in the same row in Table 2 because of the small number of coincidences), this is again not as straightforward as expected.

Some miRNAs appeared to be consistent with their known functions. For example, miR-486-5p is known to be a tumor-suppressive miRNA (see above and Text S1). As can be seen in Fig. 1, miR-486-5p was more highly expressed in normal controls. On the other hand, miR-92a was more highly expressed in cancers and other diseases, which was consistent with the previous belief that the miR-17-92 cluster is oncogenic.

Moreover, some miRNAs exhibit features contrary to previous findings. For example, miR-106b and miR-425 are believed to be oncogenic miRNAs but are expressed mainly in normal controls (Fig. 1). These apparent discrepancies may result from the measurement of miRNAs from blood samples. If we examine the PhenomiR database [34], we would find many cases in which expression in blood differs from that in tissues. For example, miR-140 is reported to be downregulated in lung cancer tissues (database IDs 132 and 134), but is overexpressed in serum from patients with lung cancer (database ID 503). miR-92a-1 is reported to be downregulated in lung cancer tissues (database IDs 530 and 543), but is overexpressed in serum from patients with lung cancer (database ID 503). These findings in blood are in agreement with those of the present study, demonstrating that miR-140 and miR-92a are expressed in the blood of lung cancer patients (Table 2 and Fig. 1). Similarly, mir-92a is highly expressed in hepatocellular carcinoma (HCC), but is decreased in plasma from HCC patients compared with that from healthy donors [35].

One may still wonder whether miRNAs in the blood can function as useful biomarkers in spite of these disagreements with tissue miRNAs. However, there are many studies that have reported inconsistencies between miRNAs in the blood and tissue miRNAs; these studies have still concluded that miRNAs in the blood can function as useful biomarkers [36]–[40]. For more detailed discussions of these studies, see 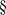
3 Frequent disagreement between blood and tissue miRNAs in Text S2.

### KEGG Pathway Analysis for miRNA Target Genes

Although a substantial number of studies have supported that the miRNAs selected in this paper are associated with several cancers and diseases, relating these miRNAs with specific diseases directly and biologically would be a more effective approach. One such method is to check whether any KEGG pathways were enriched with sets of miRNA target genes. As can be seen in the following results, our findings were validated as biologically meaningful. Up- or downregulated sets of miRNAs are selected from Table 2 and uploaded them onto DIANA-mirPath.

#### Cancer-related pathways

Some pathways directly related to specific cancers were included in the KEGG pathways. For these cancers and cancer-related diseases, it was not difficult to validate whether the up- or downregulated miRNA target genes are related to cancer. In Table 5, we list target-gene enrichment of KEGG pathways annotated as cancers investigated in our study (Table 2). For lung cancer, ductal pancreatic cancer, pancreatitis, other pancreatic tumors and diseases, prostate cancer, and melanoma, corresponding cancer-specific pathways were enriched with miRNA target genes that were up- or downregulated between patients with cancer or cancer-related diseases and healthy controls. Thus, our selection of miRNAs as biomarkers in this study was biologically validated.

**Table 5:** Cancer-specific KEGG pathways enriched in miRNA target genes.

		DIANA-mirpath		Starbase	
		–log_10_ *P*		adjusted *P*-value (<0.1)	
		up	down	up	Down
KEGG ID	description	Lung cancer			
hsa05223	Non-small cell lung cancer	3.55	7.31	4.26e-02	–
hsa05222	Small cell lung cancer	2.9	3.64	–	–
hsa05200	Pathways in cancer	–	–	–	5.69e-02
KEGG ID	description	Ductal pancreatic cancer			
hsa05212	Pancreatic cancer	9.71	4.66	1.12e-02	–
hsa05200	Pathways in cancer	–	–	1.10e-02	–
KEGG ID	description	Pancreatitis			
hsa05212	Pancreatic cancer	13.28	12.36	2.14e-05	1.10e-02
hsa05200	Pathways in cancer	–	–	5.11e-05	1.27e-03
KEGG ID	description	Other pancreatic tumors and diseases			
hsa05212	Pancreatic cancer	11.93	15.51	2.20 e-04	1.01e-03
hsa05200	Pathways in cancer	–	–	3.67 e-05	2.90e-03
KEGG ID	description	Prostate cancer			
hsa05200	Pathways in cancer	–	1.53e-04	1.44e-02	
hsa05215	Prostate cancer	8.12	8.98	–	4.04e-04
KEGG ID	description	Melanoma			
hsa05218	Melanoma	6.32	9.21	–	–

A list of cancer-specific KEGG pathways enriched in up- and/or downregulated miRNA target genes between normal controls and corresponding cancer patients. DIANA-mirpath gave 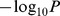-values while Starbase gave adjusted 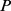-values.

#### Other pathways

Although there were no other KEGG pathways directly related to diseases, many previously known disease-related pathways are enriched with miRNA target genes. For more details about KEGG pathway enrichments related to other diseases, i.e., ovarian cancer, gastric cancer, chronic obstructive pulmonary disease (COPD), acute myocardial infraction, Wilm’s tumor, and periodontitis, see 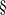
2 KEGG pathway analysis in Text S2.

### Stability

In order to confirm the findings above, i.e., commonness of miRNAs that can discriminate patients with cancers or other diseases from healthy controls, we evaluated the stability of the selection (for methodological details, see Materials and Methods).

The concept of stability was defined as follows:

*Suppose we have a set of samples, generate subsamplings many times, and apply feature extraction to each subsampling. Stability is defined as the amount of overlapping features over all subsamplings.*

If there are no features selected for all subsamplings, we can define stability alternatively by the average frequency that each feature is selected. The importance of this concept was not recognized until very recently. Abeel *et al* [14], pointed out this issue and proposed a new method that grants better performance regarding stability, RFE, and ensemble RFE. However, their method still requires classification information as prior knowledge. Earlier, Varshavsky *et al* [29] described UFF, to our knowledge, the first classification-free feature extraction method.

Thus, the concept of stability was not newly introduced by the present authors, but has already been discussed by others [14], [29]. According to our results, our implementation is the suitable that resolves the difficulty of stability of feature extractions.

Although UFF and our method are sampling-independent, as mentioned above, we checked the stability of our method for discrimination between diseased individuals and healthy controls, following the methods of Abeel *et al.* [14] or Varshavsky *et al* [29]. In our case, there were 14 diseases represented in the samples. Thus, we attempted to discriminate the normal control from each of the 14 diseases. Since 10 miRNAs were selected for each discrimination event, a total of 140 miRNAs were selected as biomarkers, although each miRNA could be selected more than once if it was selected in a different discrimination event. These 140 miRNAs could change at every subsampling. Next, we checked whether each miRNA could always be selected for all of subsamplings. If the number of miRNAs that was always selected as biomarkers was large, this means that the method was stable. We found that 8 miRNAs, i.e., hsa-miR-425, hsa-miR-15b, hsa-miR-185, hsa-miR-92a, hsa-miR-140-3p, hsa-miR-320a, hsa-miR-486-5p, and hsa-miR-16, were always selected by our method with 100% probability as biomarkers, independent of cancers and other diseases. Hsa-miR-191 and hsa-miR-106b were selected with 100% probability as biomarkers for 9 and 5 out of 14 cancers or other diseases, respectively. In addition to this, hsa-miR-19b was selected as a biomarker with 100% probability 3 times. Thus, in total, 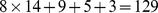 miRNAs were selected as biomarkers with 100% probability. Furthermore, all miRNAs selected as biomarkers for any cancer and diseases (Table S1) have also appeared in Table 2. Thus, it is clear that our miRNA selection was highly independent of sampling.

We also examined the stability by 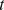-test-based feature extraction, as proposed by Keller *et al.* [13]. As expected, their results were too scattered to allow for the proposal of well-defined biomarkers consisting of 10 specific miRNAs. By our evaluation, it is very rare for an miRNA to be selected as a biomarker for cancer or other diseases with 100% probability in 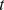-test-based feature extraction. In fact, there were only a total of 40 miRNAs selected as biomarkers with 100% probability (Table S1) in 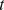-test-based feature extraction; our method identified 129 miRNAs. Based on this, it is almost a certainty that the reason Keller *et al.* [13] could not present 10 specific miRNAs as biomarkers was this heavy fluctuation. We also examined SAM, up- and downregulation by gsMMD, RFE, ensemble RFE, and UFF. We identified 30, 5, 1, 1, 0, and 111 miRNAs, respectively, that were selected as biomarkers with 100% probability (Table S1). Although UFF was as good as our novel method, UFF requires execution of SVD as many times as the number of features (in this case, the number of human miRNAs, i.e., 862), while our method requires execution of PCA only once; thus, there was no need for us to employ UFF instead of our proposed method. Therefore, it is clear that our method outperformed others from the perspective of stability of feature selection.

However, it is also true that UFF, which is the only other classification-free feature extraction method, is the second best method and is comparable to our method. This definitely demonstrates the usefulness of classification-free extraction methods for the identification of blood-borne miRNAs as biomarkers to discriminate between diseased individuals and normal controls.

### Discrimination between Diseases

One may wonder whether miRNAs can be used to discriminate not only between normal controls and diseased individuals, but also between diseases. In order to answer this question, we applied our methods to discriminate between diseases. As can be seen in Fig. S2, discrimination between diseases was also quite good. Thus, we may conclude that our methods can discriminate between diseases.

Table 6 lists the miRNAs that were used for discrimination between diseases. The miRNAs in Table 6 are almost identical to those listed in Table 2. Additionally, most of the miRNAs in Table 6 were also included in the miRNA list in Table 2. The miRNAs that were included in Table 6 but were not included with the miRNAs in Table 2, i.e., miR-103, miR-22, and miR-720, were selected only twice each among the total of 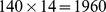 selections (see Table S2). Thus, the miRNAs selected for the discrimination between patients with cancers or other diseases and healthy controls can also discriminate between diseases well, except for discrimination between closely related diseases, e.g., pancreatitis, ductal pancreatic cancer, and other pancreatic tumors and diseases, or lung cancer and melanoma, etc (for details, see Fig. S3 and Table S3).

**Table 6:** miRNAs used for discrimination between diseases.

	1	2	3	4	5	6	7	8	9	10	11	12	13	14
Lung cancer	140-3p	15b	16	185	191	19b	30d	320a	425	486-5p	92a			
Multiple selerosis	106b	140-3p	15b	16	185	191	19b	30d	320a	425	486-5p	92a		
Other pancreatic tumors and diseases	106b	140-3p	15b	16	185	191	19b	320a	425	486-5p	92a			
Pancreatitis	106b	140-3p	15b	16	185	191	19b	30d	320a	425	486-5p	92a		
Ductal pancreatic cancer	106b	140-3p	15b	16	185	191	19b	320a	425	486-5p	92a			
Gastric cancer	106b	140-3p	15b	16	185	191	19b	22	30d	320a	425	486-5p	92a	
Sarcoidosis	140-3p	15b	16	185	191	19b	30d	320a	425	486-5p	720	92a		
Melanoma	140-3p	15b	16	185	191	19b	30d	320a	425	486-5p	92a			
Wilm’s tumor	103	106b	140-3p	15b	16	185	191	19b	30d	320a	425	486-5p	720	92a
Prostate cancer	106b	140-3p	15b	16	185	191	19b	30d	320a	425	486-5p	92a		
Acute myocardial infarction	106b	140-3p	15b	16	185	191	19b	30d	320a	425	486-5p	92a		
Periodontitis	15b	185	140-3p	320a	486-5p	16	92a	425	106b	191	19b	103	30d	
Ovarian cancer	106b	140-3p	15b	16	185	191	19b	22	30d	320a	425	486-5p	92a	
COPD	106b	140-3p	15b	16	185	191	19b	30d	320a	425	486-5p	92a		

Each row lists the miRNAs used for discrimination between the diseases. A set of 10 miRNAs among the miRNAs listed in each row was used for discrimination between the disease shown in the left most column and any of other 13 diseases or normal control. Since 10 miRNAs were selected for each of 14 discrimination analyses, a total of 140 miRNAs were selected as biomarkers. However, there are at most 14 miRNAs listed in each row. In addition to this, miRNAs shown in each row overlapped significantly with each other. This means that miRNAs to be used as biomarkers to discriminate between diseases are highly disease-independent. More detailed information about which 10 miRNAs discriminated between each pair of diseases or control/disease can be found in Table S2. All miRNAs excluding the mRNAs underlined are also in Table 2.

### Why did PCA-based Feature Selection Work So Well?

Finally, we would like explain why PCA-based feature extraction could select biomarkers that could be used to discriminate patients with cancers or other diseases from healthy controls, or to discriminate between patients with distinct diseases, without the knowledge of classification. As can be seen in the Materials and Methods, our PCA-based feature extraction did not consider classification information, even in the training set. One may find this odd because biomarkers should represent the maximum difference between more than 2 distinctive groups. The selection of useful biomarkers should not be possible without the knowledge of classification. However, from our point of view, sample selection itself contains important information about the maximum difference between distinctive groups. If we attempt to gather as many samples as possible belonging to each considered and distinctive group, any features not directly related to classification should be averaged out. For example, if one does not consider gender at all, the male to female ratio should converge to 1 to 1 when a large enough number of samples is collected for each group. This should hold true for any other feature not apparently considered. When PCA was applied to the data set in this situation, the maximum distinctive feature detected should be generated by considering any classification, as the others should have been phased out. We believe that this is the reason our PCA-based feature extraction could detect biomarkers well enough to discriminate between patients with cancers or other diseases and healthy controls in spite the lack of classification information being considered explicitly.

One may wonder whether other unsupervised clustering methods, e.g., hierarchical clustering and k-means, could have worked as well. However, for these methods, well-defined clusters must exist. This requirement is not always fulfilled. For example, although we tried to apply the feature extraction methods proposed by Chaussabel *et al* [4], [5] and based on k-means clustering of transcriptome, which was successfully applied for their purposes, we could not get enough clusters within each disease (at most 10 clusters, often only a few). That is, k-means failed to converge when a large number of clusters of miRNAs was assumed. This prevents us from effectively applying the method of Chaussabel *et al* to our data set of miRNAs. From this point of view, PCA, which does not require any clustering, is better than other unsupervised clustering methods.

### Validation Analysis

In order to demonstrate that our proposed method is not specific to present data set treated here, we added two small scale validation analyses for independent data sets (See Tables S10 and S11 in Text S4). The good performance in the validation analysis suggests the robustness of our methodology.

### Conclusion

In this paper, we proposed a new feature extraction method based on PCA for biomarker identification from miRNAs in the blood. With simulation data, our method outperformed conventional methods in detecting informative components from a mixture of informative components and noise. When our method was applied to miRNA expression in the blood of patients with cancers or other diseases and normal controls, we identified 10 common miRNAs independent of the cancer or other disease considered. PCA-based LDA using these 10 miRNAs could discriminate patients with cancers or other diseases from healthy controls as well as or slightly better than discrimination using 10 miRNAs selected by 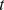-test-based feature selection. We have shown for the first time that the most distinctive feature of cancers and other diseases was not the expression of specific miRNAs, but that of common miRNAs in a cancer- or disease-specific manner.

However, this conclusion may change if more samples are considered and with cost of any technology coming down and the highthroughputs methods getting smaller to fit benchtops, detecting 100s of miRNA biomarkers identified through miRNAome studies might be much efficient and cost effective clinical application soon.

## Supporting Information

Figure S1 **Heat map of miRNA expression for miRNAs shown in** **Table 2** **.** Heat map of miRNA expression for miRNAs selected to discriminate between patients with diseases or cancers and normal controls. No miRNAs were shown to be specific to any disease. Thus, it is clear that we need a combination of miRNAs to discriminate between controls and patients with cancers or other diseases.
(EPS)

Figure S2
**Percentages of performances shown in Fig. S3.** Percentages of discriminant performances (i.e., either of precision, sensitivity, specificity or accuracy shown in Fig. S3) for all pairs of diseases and pairs of normal controls and diseases. The total number of pairs was 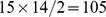. Percentages are based on the classifications, greater than 0.9 (magenta), between 0.9 and 0.8 (blue), 0.8 and 0.7 (cyan), 0.7 and 0.6 (green), 0.6 and 0.5 (yellow) and less than 0.5 (red).(EPS)

Figure S3 **Performances of discrimination between diseases using the optimal number of PCs.** Accuracy, sensitivity, specificity, and precision of each discrimination between diseases using the optimal number of PCs. The 15 columns correspond to, from left to right, lung cancer, control, multiple sclerosis, other pancreatic tumors and diseases, pancreatitis, ductal pancreatic cancer, gastric cancer, sarcoidosis, melanoma, Wilm’s tumor, prostate cancer, acute myocardial infarction, periodontitis, ovarian cancer, and COPD. Actual values can also be found in Table S3.
(EPS)

Table S1
**Frequency of miRNAs selected by several feature selections.** Frequency of miRNA selection within 90% sampling by feature selection based on PCA (100), 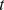-test (100), SAM (100), gsMMD_up (100), gsMMD_down (100), RFE (100), RFE ensemble (100), and UFF (100). Numbers in parentheses are the numbers of subsamplings. Cells filled with “100” indicate that the miRNA was always selected by feature extraction for discrimination between patients with the disease denoted at the top of column and healthy controls.(XLSX)

Table S2 **Frequency of miRNAs selected for discrimination between diseases.** The pair discriminated is the intersection of the table and column names, e.g., if an miRNA was selected by feature extraction for discrimination between lung cancer and control, 1 was substituted in the cell located in the row named for the miRNA and in the column named as the control in the table named as “lung cancer”.
(XLSX)

Table S3 **Performance of discrimination between diseases using the optimal number of PCs.** Accuracy, specificity, sensitivity, and precision of discrimination between diseases using the optimal number of PCs. The pair discriminated is the intersecting table and row names, e.g., the accuracy in the row named control in the table named “lung cancer” is the accuracy between control and lung cancer.
(XLSX)

Text S1 **Previous reports describing miRNAs selected by the proposed feature selection method.** Selected papers/reports describing the relationship between the miRNAs reported here and several diseases. PubMed IDs, if available, are reported with brief descriptions of the findings.
(PDF)

Text S2 **Supplementary analysis.** Detail discussions about simulation, disagreement between tissue miRNA and miRNAs in blood, and KEGG pathway analysis. Fig. S4 and Tables S4 to S9 are included in Text S2.
(PDF)

Text S3 **R code.** R code that generates simulation data used in this study.
(R) Click here for additional data file.

Text S4 **Validation Analyses.** Two small scale validation analyses using two independent data sets. It includes Tables S10 and S11. **Table S10, Validation analysis for breast cancer.** Validation of our method using independent sets for breast cancer. **Table S11, Validation analysis for carcinoma** *in situ* **/squamous cell carcinoma.** Validation of our method using independent sets for carcinoma *it in situ*/squamous cell carcinoma.
(PDF)
