# Supplementary material for: Principal Component Analysis Based Feature Extraction Approach to Identify Circulating microRNA Biomarkers
Source: PLoS One. 2013 Jun 24;8(6):e66714. doi: 10.1371/journal.pone.0066714 (PMC3715582; doi:10.1371/journal.pone.0066714)
Supplement: Text S1 — Previous reports describing miRNAs selected by the proposed feature selection method. Selected papers/reports describing the relationship between the miRNAs reported here and several diseases. PubMed IDs, if available, are reported with brief descriptions of the findings. (PDF) [file pone.0066714.s007.pdf]

Supplementary Document

Previously reported relationships with cancers/diseases for selected miRNAs

Here is a list about evidences of relationships with cancers/diseases for selected miRNAs as biomarkers to discriminate them from healthy control. This is not a comprehensive list but one mainly restricted to recent publications. Due to numerous number of literatures found, we have shown only pubmed IDs (if it is not available, DOI) as section names for citation references.

0.1 miR-425

0.1.1 20429937

miR-425 is reported as one of six most stably expressed miRNAs in both high-throughput and lower throughput RT-qPCR colorectal miRNA studies.

0.1.2 21743970

miR-425 is reported as one of theirteen miRNAs which were deregulated in two esophageal carcinoma cell lines after the treatment with cisplatin or 5-fluorouracil.

0.1.3 21055156

miR-425 is reported as one of five differentially expressed miRNAs (false discovery rate < 0.05) which were found to be associated significantly with the lymphatic metastasis of laryngeal squamous cell carcinoma ($P < 0.05$).

0.1.4 18765229

miR-425 is reported as one of three miRNAs which are up-regulated in non-stem (CD133-) cell compared with Glioblastoma stem (CD133+).

0.1.5 10.4046/trd.2009.67.5.413

miR-425 is reported to be one of twelve miRNAs which are downregulated in lung cancer.

0.1.6 20467480

miR-425-5p is reported to be expressive only in early tumors but not in late tumors.

0.2 miR-15b

0.2.1 22045185

miRNA-15b is reported to be induced with E2F-controlled genes in HPV-related cancer.

0.2.2 21725369

miR-15b is reported to regulate chemotherapy-induced epithelial-mesenchymal transition in human tongue cancer cells by targeting BMI1.

0.2.3 21454377

miR-15b is reported to be regulated by E2F1, which is a critical downstream target of the tumor suppressor retinoblastoma (RB). Thus, miR-15b is plausibly a tumor supresser.

0.2.4 21407181

miR-15b is reported to be one of nine miRNAs which are expressed in Barrett's esophagus paients.

0.2.5 21390519

miR-15b is reported to be downregulated in tumors compared with normal colorectal samples (all $P < 0.01$).

0.2.6 20404092

miR-15b is reported to be controlled by E2F which is likely to play multiple roles in cell proliferation and in proliferative diseases such as cancer.

0.2.7 20154725

miR-15b is reported to target RECK whose mutant lacking the target sites for miR-15b show augmented tumor/metastasis-suppressor activities.

0.2.8 19956871

miR-15b is reported to be the highest ranked and negatively correlated with recurrence of hepatocellular carcinoma.

0.2.9 19830692

High expression of miR-15b is reported to be significantly associated with poor recurrence free survival and overall survival by univariate Kaplan-Meier and multivariate Cox analyses in malignant melanoma.

0.2.10 19825969

miR-15b is reported to be significantly ($P < 0.05$) affected by diet x carcinogen interactions at an early stage of cancer progression (10 week post azoxymethane, a colon-specific carcinogen, injection).

0.2.11 19749800

When acute promyelocytic leukemia (APL) patients are treated with all-trans-retinoic acid (ATRA) and chemotherapy, miR-15b is reported to be one of six upregulated miRNAs.

0.2.12 19135980

miRNA-15b regulates cell cycle progression by targeting cyclins in glioma cells.

0.2.13 22096249

miR-15b is reported to be silenced via histone deacetylases in chronic lymphocytic leukemia.

0.3 miR-185

0.3.1 21962230

miR-185 targets the DNA methyltransferases 1 and regulates global DNA methylation in human glioma.

0.3.2 21832077

A miR-185-3p inhibitor rescued c-Myc expression by endogenous miR-185-3p.

0.3.3 21600139

miR-185 is one of 25 miRNAs which are down-regulated in the radioresistant cell line KYSE-150R.

0.3.4 21586611

Polymorphic nucleotide variations of miR-185 is reported to be in breast tumor.

0.3.5 21573504

miR-185 deregulation is associated with overall survival and metastasis in colorectal cancer.

0.3.6 21541354

miR-185 is reported to inhibit the growth of melanoma xenografts when implanted in SCID-NOD mice.

0.3.7 21343391

miR-185 is reported to be one of 13 miRNAs which are validated by 3'UTR-binding assays to regulate the long 3'UTR of Androgen receptor, which is expressed in all stages of prostate cancer progression, including in castration-resistant tumors.

0.3.8 21186079

miR-185 is reported to inhibit the proliferation potential of human colorectal cells.

0.3.9 20620595

The miRNA miR-185* is reported to be significantly upregulated in lung squamous cell carcinoma ($P < 0.05$), compared with normal lung tissues.

0.3.10 20603620

miRNA-185 is reported to suppress tumor growth and progression by targeting the Six1 oncogene in human cancers.

0.3.11 20420713

miR-185 is reported to be one of five miRNAs increased levels of which causes loss of function of tumor suppressors LRRC2, PTPN13, SFRP1, ERBB4, and (SLC12A1, TCF21) respectively.

0.3.12 21475928

miR-185 is reported to be one of 22 miRNAs which are significantly up-regulated in gastric cancer ($P < 0.05$).

0.3.13 19688090

miR-185 is reported to be able to induce cell cycle arrest in human non small cell lung cancer cell lines.

0.3.14 17826655

miR-185 is reported to be one of ten human miRNAs which are significantly up-regulated in bladder cancers ($P < 0.05$) compared to normal bladder mucosa and one of four miRNAs which are significantly up-regulated in renal cell carcinoma ($P < 0.05$) compared to normal kidney.

0.3.15 19688090

miR-185 can induce cell cycle arrest in human non small cell lung cancer cell lines.

0.3.16 19688090

Introduction of synthetic miR-185 is reported to suppress growth of the human non-small cell lung cancer cell lines.

0.4 miR-92a

0.4.1 22303306

Circulating miR-92a can discriminate colorectal cancer from healthy controls

0.4.2 22158052

miR-92a is reported to be one of miRNAs which are significantly increased in hemolyzed specimens (20-30 fold plasma increase; $p < 0.0000001$).

0.4.3 22043236

miR-92a is reported to be involved in von Hippel-Lindau (VHL) tumor suppressor silencing.

0.4.4 21930727

miR-92a is reported to be expressive significantly higher in colorectal cancer tissues compared with their adjacent normal tissues ($p < 0.0001$).

0.4.5 21922590

miR-92a is reported to be one of three miRNAs whose upregulation is associated with the mutation KRAS, v-Ki-ras2 Kirsten rat sarcoma viral oncogene homolog, is colorectal carcinoma tissue compared with normal colon tissue.

0.4.6 21883694

miR-92 is reported to be a key oncogenic component of the miR-17-92 cluster in colon cancer.

0.4.7 21826996

miRNA-92a is reported to be one of 8 miRNAs which are up-regulated in colon cancer.

0.4.8 21572098

miRNA-92a is reported to target and to inhibit the expression of tumor suppressor DICKKOPF-3 (DKK3) in neuroblastoma.

0.4.9 21551242

miR-92a is reported to be significantly upregulated in colorectal cancer patients in two of the plasma-based studies and in CRC tissue in one of the tissue-based studies.

0.4.10 21383985

Plasma miR-92a values in non-Hodgkin's lymphoma is reported to be extremely low ($< 5\%$), compared with healthy subjects ($P < .0001$). It could be a novel biomarker not only for diagnosis but also for monitoring lymphoma patients after chemotherapy.

0.4.11 20518884

miR-92a is highly expressed in hepatocellular carcinoma, but miR-92a in the plasmas from HCC patients is decreased compared with that from the healthy donors.

0.5 miR-140-3p

0.5.1 21890451

miR-140-3p is reported to be one of 5 miRNAs which distinguish squamous cell carcinoma (SCC)from normal lung tissues (downregulated in SCC, in their Supplementary Table 2).

0.5.2 19846888

The altered expression of miR-140-3p at 16q22 is reported to be significantly associated with several allelic imbalances or loss of heterozygosity in multiple myeloma.

0.5.3 20528768

hsa-miR-140-3p is reported to be differentially expressed (control compared with coronary artery disease, P=0.017) in whole blood.

0.5.4 20676061

miR-140-3p is reported to be one of miRNAs identified in Dupuytren's contracture samples.

0.5.5 21300873

Ratios of miR-140-3p to some other miRNAs in plasma are used to discriminate lung cancer patients from normal control.

0.5.6 21789031

miR-140-3p is reported to be upregulated compared with control/cell line (Table 1) in Osteosarcoma.

0.5.7 22052540

miR-140-3p is reported to be one of 48 miRNAs which are significantly down-regulated relative to normal endometria in both endometrial endometrioid adenocarcinomas and endometrial serous adenocarcinomas (Fig. 1A).

0.6 miR-320a

0.6.1 21743970

miR-320a is reported as one of thirteen miRNAs which were deregulated in two esophageal carcinoma cell lines after the treatment with cisplatin or 5-fluorouracil.

0.6.2 22134529

miR-320a is reported to inhibit tumor invasion by targeting neuropilin 1 and is associated with liver metastasis in colorectal cancer.

0.6.3 22120965

miR-320a is reported to be one of miRNAs which are significantly increased in stable chronic systolic heart failure patients.

0.7 miR-486-5p

0.7.1 22303306

Circulating miR-486 is associated with overall survival in non-small cell lung cancer.

0.7.2 22303306

Together with circulating miRs-21,216,210, circulating miR-486 can discriminate stage I non-small cell lung cancer from healthy controls.

0.7.3 18765229

miR-486 is reported as one of three miRNAs which is up-regulated in non-stem (CD133-) cell compared with Glioblastoma stem (CD133+).

0.7.4 21407181

miR-486-5p is reported to be one of nine miRNAs which are expressed in Barrett's esophagus paients.

0.7.5 22158052

miR-486-5p is reported to be one of miRNAs which are significantly increased in hemolyzed specimens (20-30 fold plasma increase; $p < 0.0000001$).

0.7.6 21890451

miR-486-5p is reported to be one of 5 miRNAs which distinguish squamous cell carcinoma from normal lung tissues (downregulated in SCC, in their Supplementary Table 2).

0.7.7 21864403

miR-486-5p is reported to have lower expression level in patients with malignant solitary pulmonary nodules (SPNs), as compared to subjects with benign SPNs and healthy controls (all $P \leq 0.001$).

0.7.8 20881268

miR-486-5p is more abundant in K-ras-mutated samples with respect to wild-type ones (Wilcoxon test, $P < 0.05$) in human colorectal cancer cell line.

0.7.9 20806854

miR-486-5p is reported to be one of 24 downregulated miRNAs in laryngeal cancer.

0.7.10 19946373

miR-486-5p is reported to be downregulated in 8 different tumor types (breast, colon, liver, lung, lymphoma, ovary, prostate and testis).

0.7.11 21300873

mir-486 is reported to be one of four miRNAs which are downregulated in lung cancer.

0.8 miR-16

0.8.1 23574937

Plasma miR-16 is decreased in castration-resistant metastatic prostate cancer.

0.8.2 20429937

miR-16 is reported as one of six most stably expressed miRNAs in both high-throughput and lower throughput RT-qPCR colorectal miRNA studies.

0.8.3 21055156

miR-16 is reported as one of five differentially expressed miRNAs (false discovery rate < 0.05) which were found to be associated significantly with the lymphatic metastasis of laryngeal squamous cell carcinoma ($P < 0.05$).

0.8.4 21454377

miR-16 is reported to be regulated by E2F1, which is a critical downstream target of the tumor suppressor retinoblastoma (RB). Thus, miR-16 is plausibly a tumor suppressor.

0.8.5 21390519

miR-16 is reported to be downregulated in tumors compared with normal colorectal samples (all $P < 0.01$).

0.8.6 20404092

miR-16 is reported to be controlled by E2F which is likely to play multiple roles in cell proliferation and in proliferative diseases such as cancer.

0.8.7 20154725

miR-16 is reported to target RECK whose mutant lacking the target sites for miR-16 show augmented tumor/metastasis-suppressor activities.

0.8.8 19749800

When acute promyelocytic leukemia (APL) patients are treated with all-trans-retinoic acid (ATRA) and chemotherapy, miR-16 is reported to be one of six upregulated miRNAs.

0.8.9 22158052

miR-16 is reported to be one of miRNAs which are significantly increased in hemolyzed specimens (20-30 fold plasma increase; $p < 0.0000001$).

0.8.10 22112324

miR-16 is reported to be one of 28 miRNAs which are significantly positively correlated with the time to progression of disease after cisplatin/fluorouracil chemotherapy of 82 cancer patients ($P < 0.05$).

0.8.11 22096249

miR-16 is reported to be silenced via histone deacetylases in chronic lymphocytic leukemia.

0.8.12 22049153

The mRNA stability factor HuR inhibits miR-16 targeting of Cyclooxygenase-2, which is commonly expressive in colorectal cancer. These findings provide a new explanation for tumor-derived loss of miR-16.

0.8.13 22002311

Myc represses miR-16-1 expression through recruitment of HDAC3 in mantle cell and other non-Hodgkin B-cell lymphomas.

0.8.14 21987025

miR-16-1 is reported to be one of four miRNAs which are significantly elevated in diffuse large B cell lymphoma serum when compared with normal controls ($P < 0.05$).

0.8.15 21980368

miR-16 is reported to be linked to prostate cancer metastasis by next generation sequencing technology.

0.9 miR-191

0.9.1 21969817

Hypomethylation of the hsa-miR-191 locus causes high expression of hsa-mir-191 and promotes the epithelial-to-mesenchymal transition in hepatocellular

carcinoma.

0.9.2 21956418

miR-191 down-regulation plays a role in thyroid follicular tumors through CDK6 targeting.

0.9.3 21947487

miR-191 targets N-deacetylase/N-sulfotransferase 1 and promotes cell growth in human gastric carcinoma cell line MGC803.

0.9.4 21880514

miR-191 is reported to be one of twelve miRNAs whose expression significantly changed during the progression of prostate cancer.

0.9.5 21666078

miR-191 is reported to be increased in osteosarcoma cell lines in comparison with osteoblasts.

0.9.6 21628394

miR-191 is reported to be one of sixteen miRNAs which are upregulated in gastric carcinoma.

0.9.7 21519184

miR-191 is reported to be used for normalization in profiling cancer studies of cervical tissues.

0.9.8 21255804

miR-191 is reported to be one of four miRNAs which are related to tumor recurrence of prostate cancer.

0.9.9 21084273

An SNP (SNP34091) is identified in the 3'-UTR of MDM4 that creates a putative target site for hsa-miR-191, a microRNA that is highly expressed in normal and tumor tissues.

0.10 miR-106b

0.10.1 23472110

Circulating miR-106b is down/upregulated in breast cancer.

0.10.2 23574937

Plasma miR-106b is decreased in castration-resistant metastatic prostate cancer.

0.10.3 22303306

Circulating miR-106b can discriminate gastric cancer from healthy controls.

0.10.4 20404092

miR-106b is reported to be controled by E2F which is likely to play multiple roles in cell proliferation and in proliferative diseases such as cancer.

0.10.5 21475928

miR-106b is reported to be one of 22 miRNAs which are significantly up-regulated in gastric cancer ($P < 0.05$).

0.10.6 21984948

miR-106b is reported to be one of seven miRNAs which are up-regulatied in serum and tissue of renal cell carcinoma patients.

0.10.7 21967250

miR-106b is reported to be one of four miRNAs which are significantly up-regulated in balding papilla cells.

0.10.8 21857646

miR-106b is highly abundant in cells exhibiting CD133(+) which is expressed in hematopoietic stem cells, endothelial progenitor cells, glioblastoma, neuronal and glial stem cells, various pediatric brain tumors.

0.10.9 21816922

High levels of the miR-106b-25 microRNAs is observed in primary retinoblastomas.

0.10.10 21819631

miR-106b promotes cell proliferation via targeting tumor suppressor RB in laryngeal carcinoma.

0.10.11 21698185

miR-106b is reported to inhibit p21 in AIDS Burkitt's lymphoma and diffuse large B-cell lymphoma cases, thus providing a mechanistic role for these miRNAs in AIDS non-Hodgkin's lymphomas pathogenesis.

0.10.12 21283757

Regulation of p21 gene expression by miR-106b is assessed by 3' UTR luciferase reporter assays and transfection of specific miRNA mimics in human colon cancer.

0.10.13 21059650

Bioinformatics analysis predicted microRNA-binding sites for miR-106b in the 3'-UTR of the tissue factor transcript which is expressive in breast cancer cells.

0.10.14 20878953

miR-106b is reported to be able to override radiation-induced cell cycle arrest and cell growth inhibition points to a potential therapeutic target in certain prostate cancer cells whose radiation resistance is likely due to consistently elevated level of miR-106b.

0.10.15 20818337

miR-106b gene is prominent in several signatures and correlates strongest with high proliferation in breast cancers.

0.11 miR-19b

0.11.1 21909134

miR-19b is reported to be one of five miRNAs which are identified as major growth-sustaining micro-RNAs in cancer cell lines.

0.11.2 21722265

Enhanced expression of miR-19b at the liver invasion front is observed in colorectal liver metastases.

0.11.3 21642990

miR-19b is reported to be one of five miRNAs that are capable of promoting T-cell acute lymphoblastic leukemia (T-ALL) development in a mouse model and which account for the majority of miRNA expression in human T-ALL.

0.11.4 21406606

miR-19b is reported to be one of seven miRNAs which are linked to canonical oncogenic signaling pathways and are modulated by diet and carcinogen exposure.

0.11.5 21334455

miR-19b is reported to be one of four miRNAs which are deregulated in human prostate cancer.

0.11.6 21220470

miR-19b is reported to be one of five miRNAs which are within the miR-17-92 cluster and exhibit copy number-independent overexpression in a subset of nonamplified tumors in alveolar rhabdomyosarcoma.

0.11.7 21030819

miR-19b is reported to be one of four miRNAs which might contribute to abdominal aortic aneurysm formation.

0.11.8 20949028

miR-19b is reported to be one of six miRNAs which show a low variable pattern of expression and could be considered part of the expression pattern of the healthy gastric tissue.

0.11.9 20380575

miR-19b is reported to be one of six miRNAs which are upregulated in both M059K with a cancer-associated mutation in Exon 8 and a radiosensitive cell line M059J.

0.12 miR-30d

0.12.1 22303306

Circulating miR-30d is associated with overall survival in non-small cell lung cancer.

0.12.2 20620595

miR-30d is reported to be one of Seven human miRNAs which are found to be significantly downregulated in lung squamous cell carcinoma ($P < 0.05$), compared with normal lung tissues.

0.12.3 22114136

miR-30d is reported to be associated with poor overall survival following resubsection independent of clinical covariates.

0.12.4 22058146

The chromosomal locus harboring miR-30d is amplified in $> 30\%$ of multiple types of human solid tumors.

0.12.5 21899346

miR-30d is reported to belong to one of two clusters which are downregulated in most of the resistant Ehrlich ascites tumor cells.

0.12.6 21345725

miR-30d is reported to be one of 11 miRNAs which ware expressed differently in relapsers compared with non-relapsers in ovarian cancers.
