# Supplementary material for: Principal Component Analysis Based Feature Extraction Approach to Identify Circulating microRNA Biomarkers
Source: PLoS One. 2013 Jun 24;8(6):e66714. doi: 10.1371/journal.pone.0066714 (PMC3715582; doi:10.1371/journal.pone.0066714)
Supplement: Text S2 — Supplementary analysis. Detail discussions about simulation, disagreement between tissue miRNA and miRNAs in blood, and KEGG pathway analysis. Fig. S4 and Tables S4 to S9 are included in Text S2. (PDF) [file pone.0066714.s008.pdf]

Text S2

Contents

1 Simulation **1**
1.1 Results . . . 1
1.2 Simulation data set . . . 3
1.2.1 Scenario I . . . 3
1.2.2 Scenario II . . . 3
1.2.3 Scenario III . . . 3

2 KEGG pathway analysis **3**
2.1 Ovarian and gastric cancer . . . 4
2.2 Chronic obstructive pulmonary disease (COPD) . . . 4
2.3 Acute myocardial infarction . . . 4
2.4 Wilm's tumor and periodontitis . . . 4
2.5 Sarcoidosis . . . 5

3 Frequent disagreement between blood and tissue miRNAs **5**

4 Figures **6**
4.1 Figure S4 - Results of scenarios I and II . . . 6

5 Tables **7**
5.1 Table S4 - KEGG pathways enriched in miRNA target genes in ovarian cancer . . . 7
5.2 Table S5 - KEGG pathways enriched in miRNA target genes in gastric cancer . . . 7
5.3 Table S6 - KEGG pathways enriched in miRNA target genes in COPD . . . 8
5.4 Table S7 - KEGG pathways enriched in miRNA target genes in acute myocardial infraction . . . 8
5.5 Table S8 - Correlation coefficients of $-\log P$ values of KEGG pathway enrichment for Wilm's tumor and periodontitis . . . 8
5.6 Table S9 - Enrichment of TGF β and Wnt signaling pathways in sarcoidosis . . . 9

1 Simulation

1.1 Results

Based on the procedure described in §1.2 the simulation data set, N, which was the number of miRNAs, and M, which was the number of samples, were assumed to be 100 and 200, respectively, and N_1, which was the number of miRNAs with distinct expression between 2 categories, was equal to 10. First, we generated x_{ij}, which is the expression of the ith miRNA at the jth sample, 100 times for each $D, (= 0.1, 0.2, \ldots, 2.0)$ for both scenarios I and II, while D is the parameter to control the difficulty of discrimination. Performances were evaluated by averaged values over 100 cross-ensembles. Next, we attempted to discriminate between G_1 and G_2, which corresponded to 2 categories to be discriminated, by linear discriminant analysis (LDA). Five types of feature extractions were employed in this examination as follows,

1. no feature extraction (i.e., use of all miRNAs)
2. the selection of the correct N_1 miRNA
3. the selection with t-test
4. the selection with principal component analysis (PCA)
5. the selection with significance analysis of microarrays (SAM)

Cross-validations were carried out by the leave-one-out cross validation (LOOCV) method. Averaged accuracy ranged from 0.70 to 1.0 for scenario I (Fig. S4A). Thus, G_1 and G_2 could be discriminated well within this range of D values for scenario I. For scenario II, the situation was slightly more difficult (Fig. S4B). For smaller, and thus more difficult, D values, accuracy decreased to 50%. Thus, scenario II was used for cases that were more difficult than those used in scenario I. Interestingly, the LDA that used $x_{ij}, (i \leq N_1)$ outperformed both the LDA with all features and any feature extraction method. Since $x_{ij}(i < N_1)$ is the expression level at which the means and standard deviations (SDs) of G_1 differ from those of G_2, it turned out that selecting informative components only[1] was useful for improving performance. On the other hand, any feature extraction method could achieve a similar performance to those using all miRNAs. Thus, all 3 methods successfully selected useful miRNAs without losing performance. SAM is usually believed to be better than a t-test[1], but this situation was too simple for SAM to outperform the t-test. In addition, using a group of 10 miRNAs was sufficiently large to achieve the same performance as using all miRNAs.

In Figs. S4C and S4D, we illustrate how well each feature-extraction method was able to select the correct miRNAs (i.e., those with $i \leq N_1$) when D varies. We applied t-test, PCA-based, and SAM-based feature extractions for each D while generating 100 ensembles and choosing N_1 miRNAs. The results showed that the 3 feature extraction methods had very different accuracies. When D was small enough for scenario I, t-test-based feature extraction [2] could correctly select almost all informative components. In contrast, PCA- and SAM-based feature selection could not select as many informative components when $D < 1.0$. However, it should be noted that PCA- and SAM-based feature extractions still could select more than half of the informative components correctly.

When D exceeded 1.0, the PCA-based feature extraction method began to outperform t-test-based feature extraction, while the performance of SAM-based feature extraction decreased as the performance of t-test-based feature extraction decreased. The PCA-based method was better than t-test- and SAM-based methods for $D > 1$. The number of correctly selected miRNAs by t-test and SAM-based methods continuously decreased as D increased, while that selected by the PCA-based method increased. Thus, SAM was not as good as the other 2 methods for all D values investigated in this study.

Scenario II was different from scenario I. The performances of the SAM- and t-test-based methods were very similar, while the PCA-based method outperformed both of the other methods for a wide range of D values. Since we did not know which situation occurred, i.e., whether scenario I or II was employed or which D values were employed, we concluded that it was safer to employ the PCA-based method than the other 2 methods.

The results of scenario III are shown in Table S4. Since larger D_μ and smaller D_σ indicate easier-to-resolve problems, lower rows were thought to be more difficult. Excluding situations where both D_μ and D_σ were small, i.e., $D_\mu = 0.5$ and $D_\sigma = 0.5, 1.0$, PCA-based feature extraction was comparative with or better than the other 2 methods. In particular, the number of correctly selected miRNAs outperformed values from the other methods. Thus, PCA-based feature extraction should be employed when both D_μ and D_σ are not small. Additionally, cases where both D_μ and D_σ were small were generally not interesting, since smaller D_μ and D_σ indicate that miRNAs having distinct values between the 2 classes were not expressed. Usually, biomarkers are sought among expressive miRNAs. Thus, generally speaking, PCA-based feature extraction was the most effective strategy to employ.

The results also demonstrated something interesting: better accuracy of discrimination does not always guarantee superiority of feature selection. Although t-test- and SAM-based feature selection could not select as many correct miRNAs as PCA-based feature selection could, their accuracies were almost the same as that of PCA-based feature selection. Thus, we found that even if feature selection failed to select correct miRNAs, the accuracy did not decrease very much. This finding apparently contradicts the above-mentioned observation; using the correct N_1 miRNAs achieved better accuracy than using all miRNAs. However, performance was not directly related to the number of correctly selected miRNAs among N_1 miRNAs, but related to the overall signal to noise ratio. Signal parts are represented by the difference between the 2 categories and noise parts are represented by the components that do not differ between the 2 categories. Apparently, this ratio seemed to be proportional to the number of correctly selected miRNAs, but this was not always true. Some miRNAs whose expression levels differed between the 2 categories may occasionally have more noise than some miRNAs without any difference between the 2 categories. In such cases, considering miRNAs with both small differences between the 2 categories and larger noise was worse than considering miRNAs without any differences between the 2 categories but with smaller noise.

[1]However, it does not mean that usage of more informative components always results in better performance. See Results and Discussion.

This is illustrated in Table 1 in main text. When $D_\sigma = D_\mu = 2.0$, although accuracy was about 0.82 independent of the feature extraction method employed, the number of correctly selected miRNAs was highest in PCA-based feature extraction. This demonstrated that optimal selection of miRNAs for better performance was not always the better strategy to improve (i.e., have more reasonable) feature selection. One may think that these results were caused by artifacts; however, given that the above discussion is very general, the results can be considered reasonable.

This may also be the reason that feature extraction based upon the goodness of classification often varies significantly. Better classification can be achieved by selecting not only miRNAs that are more distinct between the 2 classes, but also miRNAs with less noise. The latter may be decided by chance, thus resulting in unstable feature extraction.

1.2 Simulation data set

In order to evaluate the performance of the proposed method, we generated an artificial data set for the following 3 scenarios.

1.2.1 Scenario I

In this scenario, we controlled the amount of noise to modify the difficulty of discrimination. Suppose we have 2 pseudoclinical sets, G_1 and G_2. Samples $j \le (>) \frac{M}{2}$ belong to $G_1(G_2)$. miRNAs $i \le N_1$ are supposed to have distinct values between G_1 and G_2 while others do not, as follows.

$$x_{ij} = \begin{cases} N(\mu_{i1}, D\sigma_{i1}) & j \le \frac{M}{2}, \quad i \le N_1 \\ N(\mu_{i2}, D\sigma_{i2}) & j > \frac{M}{2}, \quad i \le N_1 \\ N(\mu_0, \sigma_0) & \qquad\qquad i > N_1 \end{cases},$$

where $N(\mu, \sigma)$ is the normal distribution with a mean μ and standard deviation (SD) of σ. $\mu_{i1}, \mu_{i2}, \sigma_{i1}, \sigma_{i2}$ are derived from the uniform distribution $\in [0, 1]$. $\mu_0 = 0$ and $\sigma_0 = 1$. $D(= 0.1, 0.2, \ldots, 2.0)$ is the parameter representing how easily G_1 and G_2 are discriminated. A larger (smaller) D indicates that it is more difficult (easier) to discriminate between the 2 pseudoclinical sets.

1.2.2 Scenario II

In this scenario, we controlled the amount of difference between the 2 categories to modify the difficulty of discrimination.

$$x_{ij} = \begin{cases} N(D\mu_{i1}, \sigma_0) & j \le \frac{M}{2}, \quad i \le N_1 \\ N(D\mu_{i2}, \sigma_0) & j > \frac{M}{2}, \quad i \le N_1 \\ N(\mu_0, \sigma_0) & \qquad\qquad i > N_1 \end{cases},$$

where all the parameters are the same as in scenario I. This time, a larger (smaller) D indicates that it is easier (harder) to discriminate between the 2 pseudoclinical sets.

1.2.3 Scenario III

In addition to these 2 above scenarios, in this third scenario, we controlled both the amount of difference between the 2 categories and the amount of noise to modify the difficulty of discrimination. The purpose of this scenario was to confirm the general tendencies found in scenarios I or II where either μ or σ was modified and thus reality was somewhat limited.

$$x_{ij} = \begin{cases} N(D_\mu\mu_{i1}, D_\sigma\sigma_{i1}) & j \le \frac{M}{2}, \quad i \le N_1 \\ N(D_\mu\mu_{i2}, D_\sigma\sigma_{i2}) & j > \frac{M}{2}, \quad i \le N_1 \\ N(\mu_0, \sigma_0) & \qquad\qquad i > N_1 \end{cases},$$

where all of the parameters are the same as in scenarios I and II. The possible values of D_μ and D_σ were restricted to $0.5, 1.0, 1.5$, or 2.0 in order to avoid having too many computational points. Thus, in this scenario, there were 16 possible combinations of D_μ and D_σ.

2 KEGG pathway analysis

In this section, we would like to discuss additional pathways related to diseases that were not discussed in the main text.

2.1 Ovarian and gastric cancer

The KEGG pathway classification does not include specific pathways directly assigned to either ovarian cancer or gastric cancer. Thus, alternatively, we compared KEGG pathways enriched with miRNA target genes and KEGG pathways reported to be related to ovarian or gastric cancer in previous studies.

Crijns *et al* [3] reported KEGG pathways related to survival probabilities in ovarian cancer. Thus, we checked whether these pathways were enriched in target genes of miRNAs selected in our study (Table S4). DIANA-mirPath listed all of the KEGG pathways enriched by survival probability-related genes and attributed P-values less than 0.01 to 12 out of 17 pathways. Starbase selected the cell cycle as an enriched pathway.

Hu and Chen [4] listed 20 significant KEGG pathways based on a comparison with protein-protein interactions in gastric cancer. We checked whether these pathways were enriched in target genes of miRNAs selected in our study (Table S5). DIANA-mirPath listed 13 out of 20 KEGG pathways enriched by protein-protein interactions (PPIs) and attributed P-values less than 0.01 to 10 out of 13 pathways. Starbase selected the Wnt signaling pathway as an enriched pathway.

These above 2 results clearly demonstrated that the KEGG pathways identified to be important in ovarian and gastric cancers were enriched in target genes of miRNAs selected by our methods.

2.2 Chronic obstructive pulmonary disease (COPD)

Ezzie *et al* [5] reported miRNA and mRNA expression profiles enriched for biological pathways that may be relevant to the pathogenesis of COPD (see their Supplementary Table E4). We checked whether pathways with relatively significant P-values were enriched in target genes of miRNAs selected in our study (Table S6). A substantial number of KEGG pathways were common between our study and theirs. This suggested that miRNAs selected by our methods correctly target biologically significant genes in COPD.

2.3 Acute myocardial infarction

Erdal *et al* [6] listed 15 KEGG pathways that were altered in response to left coronary artery ligation (LCAL) in myocardial infarction (MI) ischemic and remote zones (in their Table 2[6]). We checked whether these pathways were enriched in target genes of miRNAs selected in our study (Table S7). Ten out of 15 KEGG pathways listed by Erdal *et al* were estimated to be enriched in target genes of miRNAs using DIANA-mirPath or Starbase. Thus, our selected miRNA target genes enriched KEGG pathways that were experimentally reported to be significant.

2.4 Wilm's tumor and periodontitis

Although we could not find any KEGG pathways pertaining to Wilm's tumor or periodontitis, we found studies that reported significant up- or downregulation of miRNA combinations distinct from ours for each disease. Thus, we compared KEGG pathway target gene enrichment between these studies and our present study. If enriched KEGG pathways were common, we assumed that our selection of miRNAs was not accidental.

Schmitt *et al* [7] experimentally identified 7 upregulated miRNAs (miR-766, miR-1246, miR-197, miR-224, miR-520d-3p, miR-373, and miR-1204) and 13 downregulated miRNAs (miR-20a/b, miR-144/144*, miR-106a/b, miR-18a/b, miR-93, miR-17, miR-126, miR-1305, and miR-374a) in Wilm's tumor, while our results (Table 2 in main text) identified 4 upregulated mirPath-accepting miRNAs (miR-15b, miR-92a, miR-140-3p, and miR-320) and 6 upregulated mirPath-accepting miRNAs (miR-425, miR-185, miR-486-5p, miR-16, miR-191, and miR-106b) in Wilm's tumor.

On the other hand, Xie *et al* [8] identified 10 miRNAs (miR-126, miR-20a, miR-142-3p, miR-19a, let-7f, miR-203, miR-17, miR-223, and miR-146a/b) that were increased in inflamed periodontal tissues relative to healthy tissues, while our results (Table 2 in main text) identified 3 upregulated miRNAs (miR-140-5p, miR-320a, and miR-16) and 7 downregulated miRNAs (miR-425, miR-15b, miR-185, miR-92a, miR-486-5p, miR-191, and miR-106b).

Although there was only one miRNA in common between our study and other studies, $-\log P$-values of KEGG pathway enrichments with miRNA target genes were well correlated (Table S8). Here, P-values were attributed to KEGG pathways by DIANA-mirPath. Thus, we can conclude that our selection of miRNAs as biomarkers for Wilm's tumor and periodontitis were biologically accurate.

2.5 Sarcoidosis

Crouser *et al* [9] identified significant KEGG pathways using DIANA-mirPath as well. Their conclusions were that transforming growth factor (TGF) β and Wnt signaling pathways were enriched due to differential expression of patient miRNAs versus miRNAs from normal controls in lung tissue and peripheral blood mononuclear cells, respectively. We checked whether these 2 were recognized as enriched KEGG pathways in our miRNA profiles of target genes. Interestingly, mirPath identified both, and Starbase identified the Wnt signaling pathway (Table S9). Although they were not primary enriched pathways (TGF β was the fifth most enriched pathway in Starbase using upregulated miRNAs and Wnt was the fourth most enriched pathway in DAIANA-mirPath using downregulated miRNAs), their rankings were significantly high enough considering that there are more than 250 human pathways registered in KEGG. Although only the fact that these 2 were enriched in our miRNA target genes is not enough to conclusively state that our results are consistent with theirs, we can at least conclude that our study and their study do not disagree with each other.

3 Frequent disagreement between blood and tissue miRNAs

In spite of frequent differences in miRNA expression between tissues and blood, blood miRNAs can often serve as useful biomarkers.

Wulfken *et al* [10] reported that 103 miRNAs are upregulated and 83 miRNAs are downregulated in clear cell renal cell carcinoma (ccRCC) when compared with adjacent nonmalignant tissue. They also reported that 189 miRNAs are upregulated and 9 miRNAs are downregulated in the serum of ccRCC patients when compared with healthy controls. Only 35 of these miRNAs were identified as exhibiting common expression between tissue and serum. However, 7 of these candidate miRNAs were chosen as biomarkers.

Wu *et al* [11] found different miRNA expression profiles in the serum and tissues of breast cancer patients and control subjects by next-generating sequence (NGS) - sequencing by oligonucleotide ligation and detection (SOLiD) sequencing. Only 7 miRNAs were found to be co-upregulated (i.e., miR-103, miR-23a, miR-29a, miR-222, miR-23b, miR-24, and miR-25). Out of these 7 miRNAs, they concluded that serum miR-222 was a potential biomarker that could be used to distinguish between breast cancer patients and healthy controls.

Wang and Gu [12] found that miR-29a and miR-92a are expressed in metastatic tissues but not in non-metastatic tissues, while miR-29a is expressed in the serum of patients with metastases and not in the serum of patients without metastases. This allowed them to conclude that serum miR-29a is a useful biomarker.

The above studies investigated miRNA biomarkers among miRNAs that are commonly up- or downregulated between disease/cancer patients and normal controls. However, miRNAs that do not show common up- or downregulation between tissue and serum can also be useful biomarkers.

Kurashige *et al* [13] found that serum miR-21 may be a novel biomarker in patients with esophageal squamous cell carcinoma. However, there was no significant correlation between serum and tissue miR-21 expression in their study.

Lee *et al* [14] identified 6 miRNAs that were overexpressed and 3 miRNA that were underexpressed in both tissue and serum samples from patients with squamous cell carcinoma. They also found that 16 miRNAs were overexpressed and 22 miRNA were underexpressed in both tissue and serum samples from patients with adenocarcinoma. However, fold changes substantially differed between serum and tissue samples. They proposed that microRNA-23a was a novel serum-based diagnostic biomarker for lung adenocarcinoma, with a fold change of more than 200, while that in tissue was only 3.

Thus, independent of the relationship between tissue miRNAs and serum miRNAs and the lack of explanation of these differences, tissue or serum miRNAs can be useful biomarkers. Guaranteeing that miRNA expression is common between tissue and serum is not always important for miRNAs to be useful biomarkers.

Although we cannot fully understand their features, when miRNAs from the blood are used as biomarkers to discriminate patients with cancers/diseases from healthy controls, our findings were at least consistent with a previous proposal regarding tissue miRNAs [15]; significant features do not always include expression of cancer- and/or disease-specific miRNAs, but do also include expression of common miRNAs in a cancer- and/or disease-specific manner. More investigations of these topics are necessary.

4 Figures

4.1 Figure S4 - Results of scenarios I and II

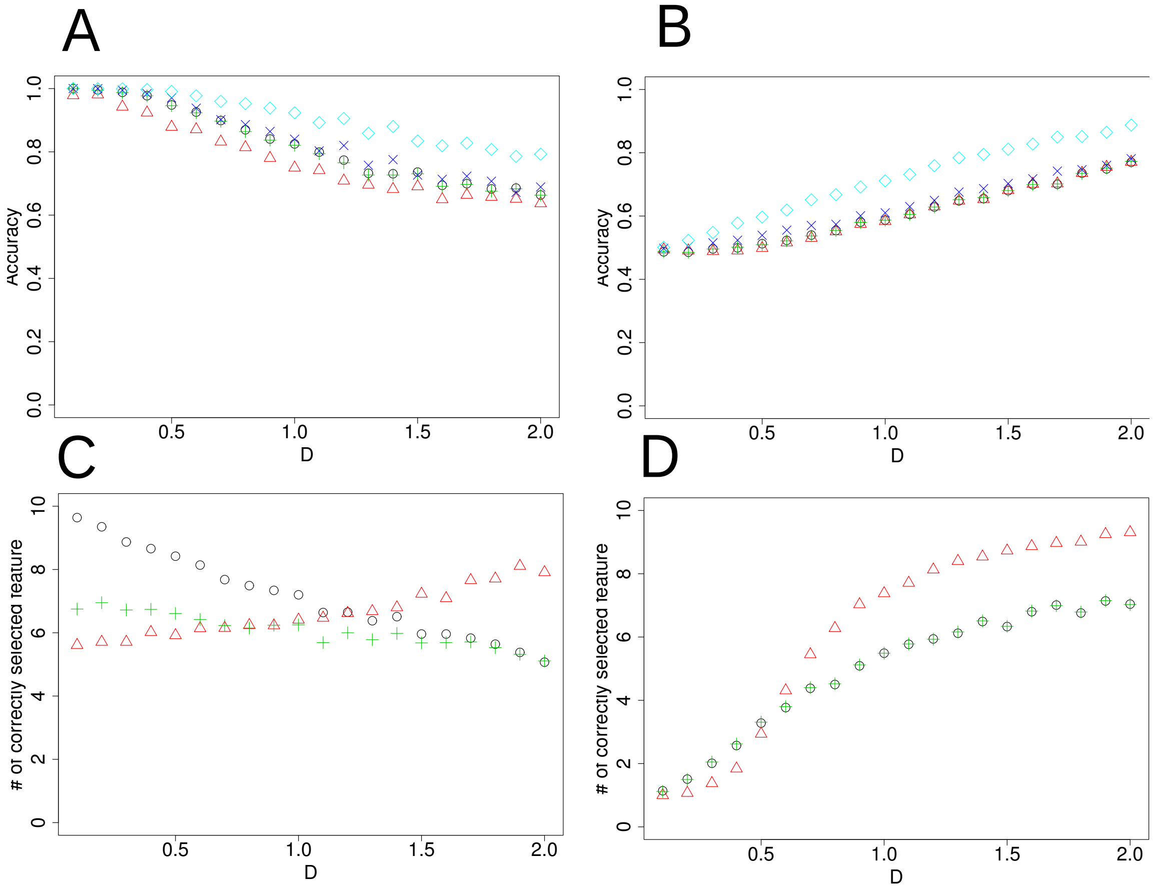

A) Accuracy of LDA between G_1 and G_2 using simulation data as a function of D (scenario I). The black $\circ$ is the result obtained using $N_1(=10)$ miRNAs selected with t-test-based feature extraction, the red $\triangle$ is from PCA-based feature extraction, the green $+$ is from SAM-based feature extraction, the blue $\times$ is from analysis using all miRNAs, and the cyan square is the result obtained using N_1 miRNAs $(i \leq N_1)$. A larger D corresponds to a more difficult classification. **B**) Accuracy of LDA between G_1 and G_2 using simulation data as a function of D (scenario II). In contrast to **A**), a larger D corresponded to an easier classification. **C**) Number of correctly selected miRNAs among the selected $N_1(=10)$ miRNAs (scenario I). **D**) Number of correctly selected miRNAs among the selected $N_1(=10)$ miRNAs (scenario II).

5 Tables

5.1 Table S4 - KEGG pathways enriched in miRNA target genes in ovarian cancer

Comparison between KEGG pathways enriched in survival probability-related genes (the first and second columns) and P-values attributed to them based on enrichment of miRNA target genes (from the third to sixth column).

		Diana-mirpath		Sterbase	
		$-\log_{10} P$		adjusted P-value (< 0.1)	
KEGG ID	description	up	down	up	down
hsa00190	Oxidative phosphorylation	5.62	4.04		
hsa00230	Purine metabolism	3.58	1.82		
hsa01430	Cell communication	0.65	0.76		
hsa04020	Calcium signaling pathway	0.23	2.72		
hsa04060	Cytokine-cytokine receptor interaction	1.61	1.61		
hsa04110	Cell cycle	3.69	8.65	3.15E-02	1.21E-02
hsa04310	Wnt signaling pathway	11.69	14.85		
hsa04360	Axon guidance	9.5	21.68		
hsa04510	Focal adhesion	13.15	16.61		
hsa04514	Cell adhesion molecules (CAMs)	1.45	0.33		
hsa04530	Tight junction	1.41	6.02		
hsa04630	Jak-STAT signaling pathway	2.45	1.65		
hsa04650	Natural killer cell mediated cytotoxicity	0.7	0.25		
hsa04670	Leukocyte transendothelial migration	1.28	1.64		
hsa04810	Regulation of actin cytoskeleton	14.19	5.58		
hsa04910	Insulin signaling pathway	8.14	5.44		
hsa04020	MAPK signaling pathway	6.42	23.17		

5.2 Table S5 - KEGG pathways enriched in miRNA target genes in gastric cancer

Comparison between KEGG pathways enriched in PPI (from the first to the third columns) and P-values attributed to them based on enrichment of miRNA target genes (from the third to sixth column).

			DIANA-mirPath		Starbase	
		P-value	$-\log_{10} P$		adjusted P-balue ($P < 0.1$)	
Pathway ID	Description	PPI	up	down	up	down
hsa00071	Fatty acid metabolism	0.0038	0.14	0.47		
hsa00280	Valine, leucine, and isoleucine degradation	0.0064	2.04	1.67		
hsa00520	Amino sugar and nucleotide sugar metabolism	0.0107	-	-		
hsa00534	Glycosaminoglycan biosynthesis " heparan sulfate	0.0301	5.79	1.99		
hsa00910	Nitrogen metabolism	0.0113	0	0.29		
hsa00980	Metabolism of xenobiotics by cytochrome P450	0.0133	2.06	3.8		
hsa03010	Ribosome	0.0438	6.93	5.55		
hsa03060	Protein export	0.0113	0.45	1.48		
hsa03420	Nucleotide excision repair	0	-	-		
hsa04012	ErbB signaling pathway	0.012	3.96	9.27		
hsa04062	Chemokine signaling pathway	0	-	-		
hsa04310	Wnt signaling pathway	0.0453	11.69	19.9		4.60E-04
hsa04510	Focal adhesion	0.0038	11.95	18.99		
hsa04740	Olfactory transduction	0.0384	0.2	0.4		
hsa04930	Type II diabetes mellitus	0.0181	5.5	1.67		
hsa04964	Proximal tubule bicarbonate reclamation	0.0145	-	-		
hsa04512	ECM-receptor interaction	0.0307	3.02	0.2		
hsa05144	Malaria	0.048	-	-		
hsa05322	Systemic lupus erythematosus	0.018	-	-		
hsa05340	Primary immunodeficiency	0.0332	-	-		

5.3 Table S6 - KEGG pathways enriched in miRNA target genes in COPD

Comparison between KEGG pathways enriched in miRNA and/or miRNA differentially expressed (from the first to the forth columns) and P-values attributed to them based on enrichment of miRNA target genes (from the fifth to eighth column). The "Basis" column indicates which target gene table prediction was used for the estimation of KEGG pathway enrichment.

			Ezzie *et al* [5]	DIANA-mirPath		Starbase	
			P-value	$-\log_{10} P$		adjucted P-value ($P < 0.1$)	
KEGG ID	Description	Basis		up	down	up	down
hsa05211	Renal cell carcinoma	TargetScan	2.77E-08	17.81	3.32	2.19e-02	-
hsa05223	Non-small cell lung cancer	TargetScan	2.60E-05	10.2	1.4	8.19e-02	-
hsa05222	Small cell lung cancer	TargetScan	5.52E-09	5.26	5.24	-	-
hsa05200	Pathways in cancer	TargetScan	1.08E-24	-	-	2.15e-04	-
hsa05212	Pancreatic cancer	TagetScan	1.11E-05	15.77	4.12	2.11e-04	-
hsa05220	Chronic myeloid leukemia	TargetScan	1.29E-08	16.12	1.65	6.46e-03	-
hsa05221	Acute myeloid leukemia	TargetScan	1.92E-06	5.63	-	2.775e-02	-
hsa05210	Colorectal cancer	TargetScan	5.52E-09	-	2.82	9.01e-02	-
hsa04114	Oocyte meiosis	TargetScan	1.95E-05	-	-	-	3.11e-02
hsa04510	Focal adhesion	TargetScan	4.11E-12	17.88	8.17	-	-
hsa04520	Adherens junction	TargetScan	1.24E-08	9.83	4.25	3.24e-04	-
hsa04310	Wnt signaling pathway	TargetScan	1.47E-14	22.64	8.48	1.25e-03	-
hsa04010	MAPK signaling pathway	TargetScan	1.87E-13	24.24	2.39	-	-
hsa04350	TGF-beta signaling pathway	TargetScan	9.71E-09	15.47	3.51	2.07e-04	-
hsa04120	Ubiquitin mediated proteolysis	miRanda	2.27E-06	10.2	5.23	5.33e-03	-

5.4 Table S7 - KEGG pathways enriched in miRNA target genes in acute myocardial infraction

Comparison between KEGG pathways in response to LCAL in MI ischemic and remote zones (from the first to the third columns) and P-values attributed to them based on enrichment of miRNA target genes (from the forth to seventh column).

		Erdal *et al* [6]	DIANA-mirPath		Starbase	
		P-value	$-\log_{10} P$		adjusted P-balue ($P < 0.1$)	
Pathway ID	Description		up	down	up	down
hsa03010	Ribosome	0.0354	5.52	-	-	-
hsa04120	Ubiquitin-mediated proteolysis	0.0309	8.94	8.79	-	-
hsa03320	PPAR signaling pathway	0.0315	0.79	1.92	-	-
hsa04810	Regulation of actin cytoskeleton	0.0432	12.44	10.6	-	-
hsa04530	Tight junction	0.0014	4.53	2.63	-	-
hsa04520	Adherens junction	0.0315	10.8	9.32	4.16e-03	2.77e-03
hsa04620	Toll-like receptor signaling pathway	0.0260	0.35	0.39	-	-
hsa05218	Cytokine-cytokine receptor interaction	0.0172	16.26	0.4	-	-
hsa04670	Leukocyte transendothelial migration	0.0063	1.58	-	-	-
hsa04510	Focal adhesion	0.0022	17.83	14.42	-	-

5.5 Table S8 - Correlation coefficients of $-\log P$ values of KEGG pathway enrichment for Wilm's tumor and periodontitis

Correlation coefficients of $-\log P$ values. P-values were computed for each KEGG pathway based on enrichment of miRNA target genes identified to be biomarkers that discriminate patients with Wilm's tumor or periodontitis from normal controls. miRNA selection was from either our study or other studies. The number indicates the number of commonly selected KEGG pathways between our study and other studies. P-values are those attributed to correlation coefficients. Cor indicates Pearson's correlation coefficient. Up and down indicate that genes targeted by upregulated and downregulated miRNAs were considered, respectively.

Wilm's tumor	Our study	Schmitt *et al* [7]	Number	Cor	P-value
	down	down	44	0.52	< 2.2E-16
Periodontitis	Our study	Xie *et al* [8]	Number	Cor	P-value
	up	up	148	0.81	< 2.2E-16
	down	up	162	0.84	< 2.2E-16

5.6 Table S9 - Enrichment of TGF β and Wnt signaling pathways in sarcoidosis

TGF β and Wnt signaling pathways in sarcoidosis, by target genes of up- and/or downregulated miRNAs detected by our selection method. The numbers in parentheses indicate the rank order of significance among all KEGG pathways.

	mirPath $-\log_{10} P$-value		Starbase adjsuted P-value	
KEGG Pathway	up	down	up	down
Wnt signaling pathway	7.09 (22th)	11.22 (4th)	-	-
TGF-β signaling pathway	8.13 (16th)	2.4 (39th)	1.05E-03 (5th)	-

References

[1] Kuo L, Yu F, Zhao Y (2008) Statistical mehods for identifying differently expressed genes in replicated microarry experiments: A review. In: Biswas A, Datta S, Fine JP, Segal MR, editors, Statistical Advances in the Biomedical Sciences, Willey-INTERSCIENCE, Wiley Series in Probability and Statistics, chapter 20. pp. 341–363.

[2] Keller A, Leidinger P, Bauer A, Elsharawy A, Haas J, et al. (2011) Toward the blood-borne miRNome of human diseases. Nat Methods 8: 841–843.

[3] Crijns AP, Fehrmann RS, de Jong S, Gerbens F, Meersma GJ, et al. (2009) Survival-related profile, pathways, and transcription factors in ovarian cancer. PLoS Med 6: e24.

[4] Hu K, Chen F (2012) Identification of significant pathways in gastric cancer based on protein-protein interaction networks and cluster analysis. Genet Mol Biol 35: 701–708.

[5] Ezzie ME, Crawford M, Cho JH, Orellana R, Zhang S, et al. (2012) Gene expression networks in COPD: microRNA and mRNA regulation. Thorax 67: 122–131.

[6] Erdal C, Karakülah G, Fermanci E, Kunter I, Silistreli E, et al. (2012) Early biventricular molecular responses to an acute myocardial infarction. Int J Med Sci 9: 74–82.

[7] Schmitt J, Backes C, Nourkami-Tutdibi N, Leidinger P, Deutscher S, et al. (2012) Treatment-independent miRNA signature in blood of wilms tumor patients. BMC Genomics 13: 379.

[8] Xie YF, Shu R, Jiang SY, Liu DL, Zhang XL (2011) Comparison of microRNA profiles of human periodontal diseased and healthy gingival tissues. Int J Oral Sci 3: 125–134.

[9] Crouser ED, Julian MW, Crawford M, Shao G, Yu L, et al. (2012) Differential expression of microRNA and predicted targets in pulmonary sarcoidosis. Biochem Biophys Res Commun 417: 886–891.

[10] Wulfken LM, Moritz R, Ohlmann C, Holdenrieder S, Jung V, et al. (2011) MicroRNAs in renal cell carcinoma: diagnostic implications of serum miR-1233 levels. PLoS ONE 6: e25787.

[11] Wu Q, Wang C, Lu Z, Guo L, Ge Q (2012) Analysis of serum genome-wide microRNAs for breast cancer detection. Clin Chim Acta 413: 1058–1065.

[12] Wang LG, Gu J (2012) Serum microRNA-29a is a promising novel marker for early detection of colorectal liver metastasis. Cancer Epidemiol 36: e61–67.

[13] Kurashige J, Kamohara H, Watanabe M, Tanaka Y, Kinoshita K, et al. (2012) Serum microRNA-21 is a novel biomarker in patients with esophageal squamous cell carcinoma. J Surg Oncol 106: 188–192.

[14] Lee Y, Cho H, Lee S, Yun S, Kim J, et al. (2011) Microrna-23a: A novel serum based diagnostic biomarker for lung adenocarcinoma. Tuberc Respir Dis 71: 8-14.

[15] Bandyopadhyay S, Mitra R, Maulik U, Zhang MQ (2010) Development of the human cancer microRNA network. Silence 1: 6.
