# Supplementary material for: Principal Component Analysis Based Feature Extraction Approach to Identify Circulating microRNA Biomarkers
Source: PLoS One. 2013 Jun 24;8(6):e66714. doi: 10.1371/journal.pone.0066714 (PMC3715582; doi:10.1371/journal.pone.0066714)
Supplement: Text S4 — Validation Analyses. Two small scale validation analyses using two independent data sets. It includes Tables S10 and S11. Table S10, Validation analysis for breast cancer. Validation of our method using independent sets for breast cancer. Table S11, Validation analysis for carcinoma in situ /squamous cell carcinoma. Validation of our method using independent sets for carcinoma it in situ/squamous cell carcinoma. (PDF) [file pone.0066714.s010.pdf]

Validation analysis

Although we could not find any independent large scale and publically available data sets that are associated with targeted diseases in this study, here we present two small scale validation analyses, for reference. Although these two diseases, breast cancer and carcinoma, were not investigated in the main text, since anyway our claim was that we have identified "universal" biomarkers which can discriminate several diseases from normal control, it was not a bad idea to apply our method to discriminate these two diseases from healthy control.

Normal control vs breast cancer

Leidner ans Thompson [1] measured circulating microRNA in breast cancer. We downloaded 20 normal controls and 20 pre-resection data set from GEO:ID GSE41526. 11 miRNAs expression (miRs-425, 15b, 185, 92a, 140-3p, 320a, 486-5p, 16, 191 106b, and 19b. 30d was excluded because miR-30d was listed only once in Table 2) were extracted and discriminant analysis was performed as described in the main text. Table S10 shows the results. Accuracy was 0.77, P-value and odds ratio computed by Fisher test were 0.001 and 11, respectively. Area under the curve (AUC) under receiver operating characteristic (ROC) curve was 0.77. Thus, these two classes were well discriminated.

Table S10: Validation analysis. The number of principal components used was four.

		True	
		Control	Breast Cancer
**Predicted**	Control	16	5
	Breast cancer	4	15

Normal control vs carcinoma *in situ*/squamous cell carcinoma

Maclellan *et al* [2] measured serum miRNA in carcinoma. We have downloaded 26 normal controls and 30 pre-surgery HRL patients from GEO:ID GSE37472. 11 miRNAs expression (miRs-425, 15b, 185, 92a, 140-3p, 320a, 486-5p, 16, 191 106b, and 19b. 30d was excluded because miR-30d was listed

only once in Table 2) were extracted and discriminant analysis was performed as described in the main text. Table S11 shows the results. Accuracy was 0.80, P-value and odds ratio computed by Fisher test were 1×10^{-5} and 15.7, respectively. AUC under ROC curve was 0.82. Thus, these two classes were well discriminated.

Table S11: Validation analysis. The number of principal components used was 11.

		**True**	
		Control	Carcinoma
**Predicted**	Control	21	6
	Carcinoma	5	24

In conclusion, our proposed method was useful for two independent data sets, too. This demonstrates robustness of our method.

References

[1] Leidner RS, Li L, Thompson CL. PLoS One. 2013;8(3):e57841. doi: 10.1371/journal.pone.0057841. Epub 2013 Mar 5. Dampening enthusiasm for circulating microRNA in breast cancer.

[2] Maclellan SA, Lawson J, Baik J, Guillaud M, Poh CF, Garnis C. Cancer Med. 2012 Oct;1(2):268-74. doi: 10.1002/cam4.17. Epub 2012 Jul 19. Differential expression of miRNAs in the serum of patients with high-risk oral lesions.
